# Supplementary material for: Genomic surveillance of Clostridioides difficile transmission and virulence in a healthcare setting
Source: mBio. 2024 Feb 8;15(3):e03300-23. doi: 10.1128/mbio.03300-23 (PMC10936198; doi:10.1128/mbio.03300-23)
Supplement: Supplemental figures and tables — Fig. S1-S5; Tables S1-S3. [file mbio.03300-23-s0001.pdf]

Supplemental Material

This file includes:

- Supplemental Figure 1-5
- Supplemental Tables 1-3

-

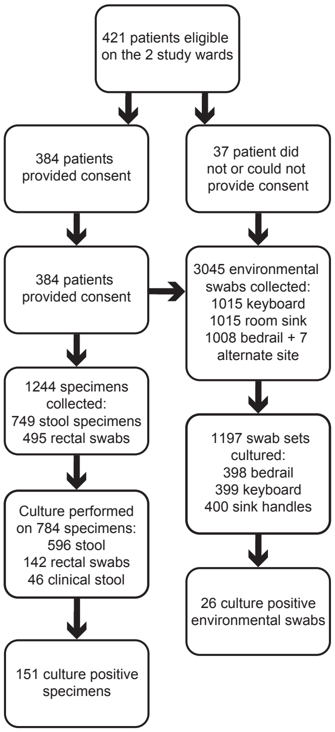

Supplementary Figure 1: Bubble plot of enrollment, collection, and culture numbers.

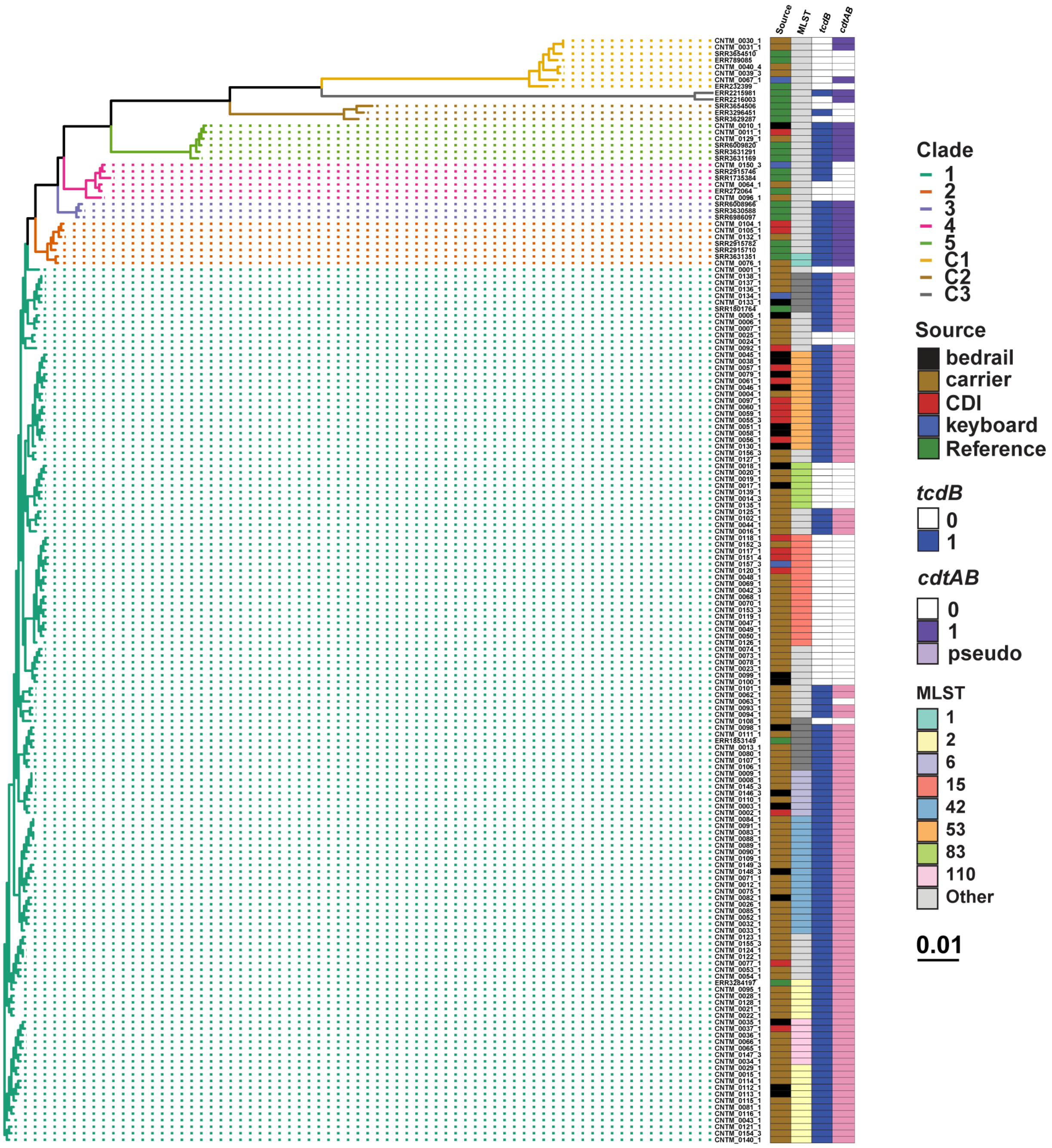

Supplementary Figure 2: Phylogenetic tree of isolates collected in this study and select references (Supplementary Table 2).

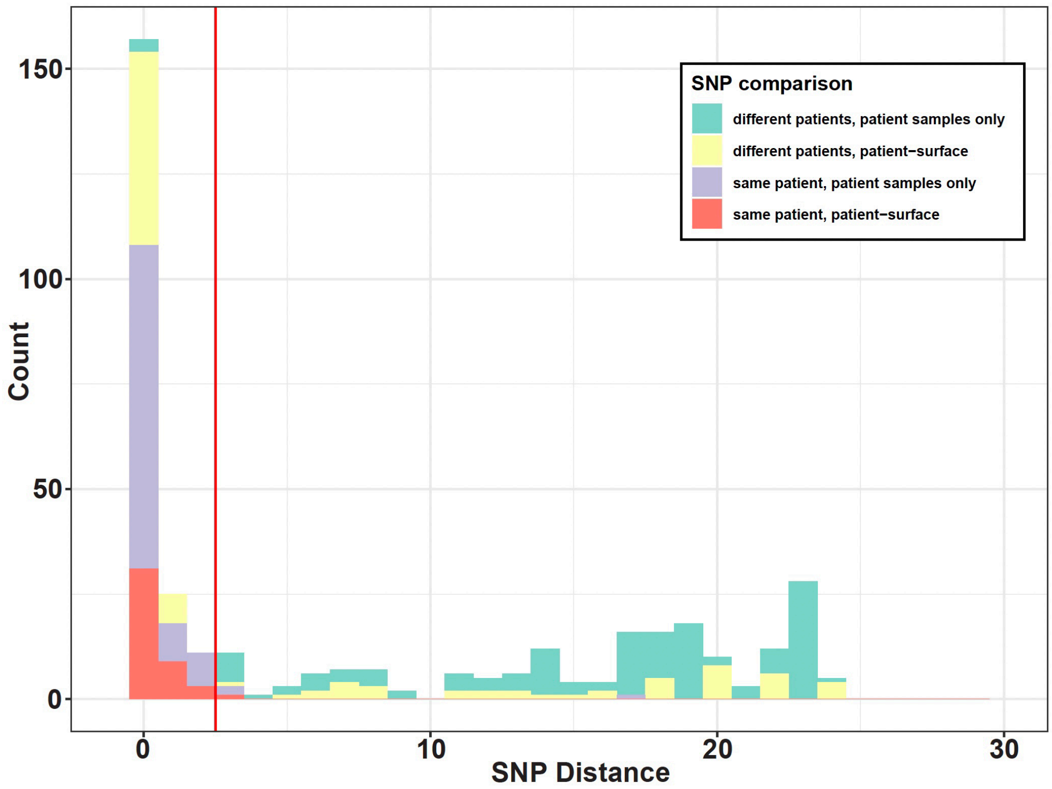

Supplementary Figure 3: Histogram of core genome SNP distances between different within-MLST isolate comparisons, zoomed to show SNP cutoff (red line at 2 SNPs).

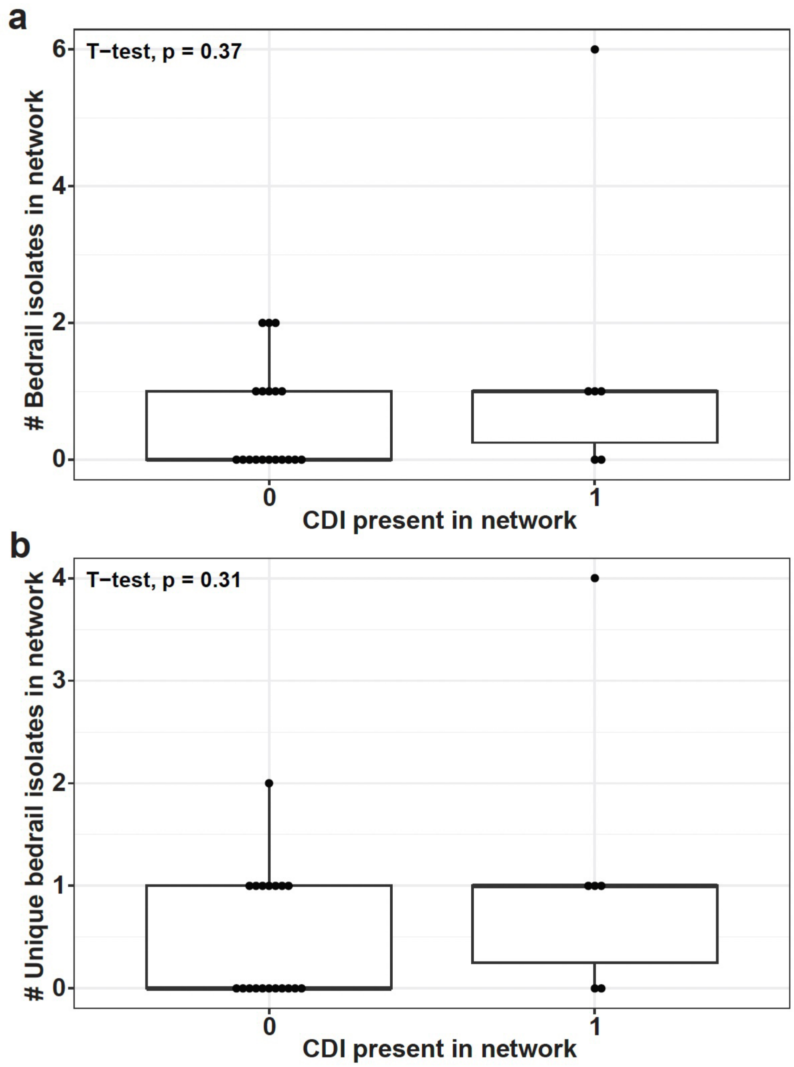

Supplementary Figure 4: A) Total number of bedrail isolates in networks either containing a CDI case or not containing a CDI case. B) Number of unique bedrails contaminated in a network either containing a CDI case or not containing a CDI case. Student’s t-test.

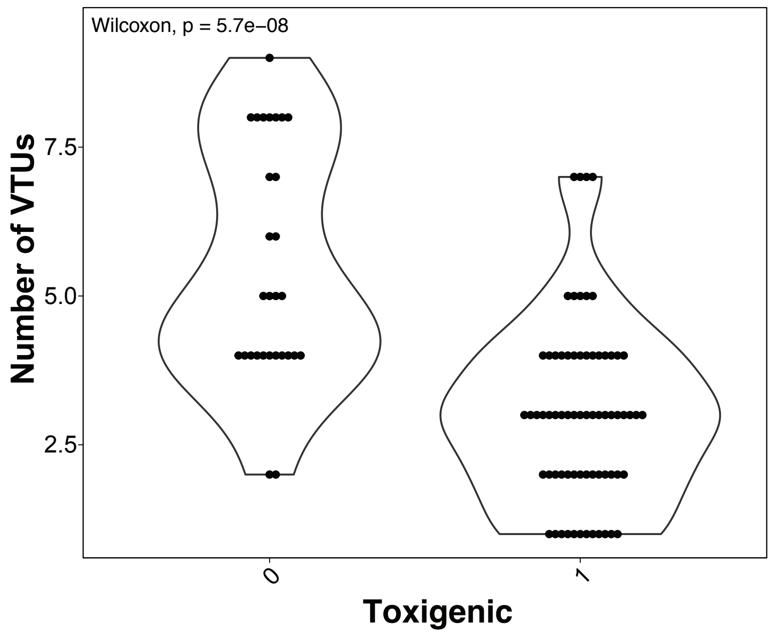

Supplementary Figure 5: Phage population richness across toxigenic and nontoxigenic isolates in our cohort. Wilcoxon test, p=5.7e-8.

Supplementary Table 1: Raw isolate metadata.

Project Name	Good?	Assembly	contigs	Highest Mash Result	Aligned Bases	Avg Identity
CNTM_0001	1	CNTM_0001_1	90	GCF_000438845.1.report	90.07	99.08
CNTM_0002	1	CNTM_0002_1	182	GCF_000438845.1.report	89.78	99.29
CNTM_0003	1	CNTM_0003_1	128	GCF_000438845.1.report	89.78	99.29
CNTM_0004	1	CNTM_0004_1	71	GCF_000438845.1.report	96.2	99.35
CNTM_0006	1	CNTM_0006_1	124	GCF_000438845.1.report	93.55	99.39
CNTM_0005	1	CNTM_0005_1	165	GCF_000438845.1.report	93.5	99.4
CNTM_0007	1	CNTM_0007_1	116	GCF_000438845.1.report	93.54	99.4
CNTM_0145	1	CNTM_0145_3	112	GCF_000438845.1.report	92.13	99.32
CNTM_0009	1	CNTM_0009_1	74	GCF_000438845.1.report	92.13	99.32
CNTM_0008	1	CNTM_0008_1	82	GCF_000438845.1.report	92.14	99.32
CNTM_0146	1	CNTM_0146_3	129	GCF_000438845.1.report	92.18	99.33
CNTM_0011	1	CNTM_0011_1	127	GCF_000438845.1.report	88.62	96.29
CNTM_0010	1	CNTM_0010_1	124	GCF_000438845.1.report	88.62	96.3
CNTM_0141	0	0	#N/A	0	0	0
CNTM_0012	1	CNTM_0012_1	70	GCF_000438845.1.report	95.28	99.33
CNTM_0013	1	CNTM_0013_1	76	GCF_000438845.1.report	94.87	99.36
CNTM_0014	1	CNTM_0014_3	135	GCF_000438845.1.report	93.01	99.25
CNTM_0015	1	CNTM_0015_1	103	GCF_000438845.1.report	90.55	99.25
CNTM_0016	1	CNTM_0016_1	107	GCF_000438845.1.report	93.28	99.2
CNTM_0017	1	CNTM_0017_1	120	GCF_000438845.1.report	92.99	99.26
CNTM_0019	1	CNTM_0019_1	152	GCF_000438845.1.report	93.02	99.26
CNTM_0018	1	CNTM_0018_1	131	GCF_000438845.1.report	92.99	99.25
CNTM_0020	1	CNTM_0020_1	133	GCF_000438845.1.report	92.97	99.27
CNTM_0021	1	CNTM_0021_1	115	GCF_000438845.1.report	94.87	99.23
CNTM_0022	1	CNTM_0022_1	115	GCF_000438845.1.report	94.9	99.24
CNTM_0023	1	CNTM_0023_1	79	GCF_000438845.1.report	92.53	99.18
CNTM_0024	1	CNTM_0024_1	110	GCF_000438845.1.report	92.58	99.17
CNTM_0025	1	CNTM_0025_1	210	GCF_000438845.1.report	92.68	99.19
CNTM_0026	1	CNTM_0026_1	371	GCF_000438845.1.report	96.15	99.37
CNTM_0027	0	0	#N/A	0	0	0
CNTM_0028	1	CNTM_0028_1	85	GCF_000438845.1.report	95.02	99.29
CNTM_0029	1	CNTM_0029_1	73	GCF_000438845.1.report	90.29	99.24
CNTM_0030	1	CNTM_0030_1	70	GCF_000438845.1.report	80.57	91.53
CNTM_0031	1	CNTM_0031_1	85	GCF_000438845.1.report	80.58	91.54
CNTM_0032	1	CNTM_0032_1	303	GCF_000438845.1.report	96.06	99.36
CNTM_0033	1	CNTM_0033_1	89	GCF_000438845.1.report	96.06	99.35

CNTM_0036	1	CNTM_0036_1	83	GCF_000438845.1.report	95.03	99.3
CNTM_0034	1	CNTM_0034_1	130	GCF_000438845.1.report	95.06	99.32
CNTM_0035	1	CNTM_0035_1	93	GCF_000438845.1.report	95.06	99.33
CNTM_0037	1	CNTM_0037_1	90	GCF_000438845.1.report	95.05	99.31
CNTM_0038	1	CNTM_0038_1	131	GCF_000438845.1.report	95.55	99.34
CNTM_0039	1	CNTM_0039_3	142	GCF_000438845.1.report	75.61	91.52
CNTM_0040	1	CNTM_0040_4	145	GCF_000438845.1.report	75.53	91.51
CNTM_0041	0	0	#N/A	0	0	0
CNTM_0042	1	CNTM_0042_3	131	GCF_000438845.1.report	92.94	99.2
CNTM_0043	1	CNTM_0043_1	163	GCF_000438845.1.report	93.73	99.26
CNTM_0044	1	CNTM_0044_1	479	GCF_000438845.1.report	94.54	99.26
CNTM_0050	1	CNTM_0050_1	407	GCF_000438845.1.report	94.28	99.27
CNTM_0047	1	CNTM_0047_1	98	GCF_000438845.1.report	94.21	99.25
CNTM_0045	1	CNTM_0045_1	98	GCF_000438845.1.report	95.63	99.36
CNTM_0048	1	CNTM_0048_1	77	GCF_000438845.1.report	94.27	99.26
CNTM_0046	1	CNTM_0046_1	106	GCF_000438845.1.report	95.55	99.35
CNTM_0049	1	CNTM_0049_1	139	GCF_000438845.1.report	94.19	99.25
CNTM_0051	1	CNTM_0051_1	199	GCF_000438845.1.report	95.62	99.35
CNTM_0052	1	CNTM_0052_1	193	GCF_000438845.1.report	96.04	99.36
CNTM_0053	1	CNTM_0053_1	473	GCF_000438845.1.report	94.01	99.33
CNTM_0054	1	CNTM_0054_1	117	GCF_000438845.1.report	94	99.32
CNTM_0055	1	CNTM_0055_3	150	GCF_000438845.1.report	95.56	99.36
CNTM_0060	1	CNTM_0060_1	103	GCF_000438845.1.report	95.55	99.35
CNTM_0056	1	CNTM_0056_1	159	GCF_000438845.1.report	95.56	99.35
CNTM_0057	1	CNTM_0057_1	82	GCF_000438845.1.report	95.57	99.36
CNTM_0061	1	CNTM_0061_1	62	GCF_000438845.1.report	95.63	99.36
CNTM_0058	1	CNTM_0058_1	218	GCF_000438845.1.report	95.57	99.36
CNTM_0059	1	CNTM_0059_1	124	GCF_000438845.1.report	95.59	99.36
CNTM_0062	1	CNTM_0062_1	121	GCF_000438845.1.report	92.33	99.11
CNTM_0063	1	CNTM_0063_1	306	GCF_000438845.1.report	91.44	99.11
CNTM_0064	1	CNTM_0064_1	253	GCF_000438845.1.report	89.17	97.98
CNTM_0065	1	CNTM_0065_1	122	GCF_000438845.1.report	94.49	99.29
CNTM_0147	1	CNTM_0147_3	277	GCF_000438845.1.report	94.7	99.3
CNTM_0066	1	CNTM_0066_1	134	GCF_000438845.1.report	94.52	99.29
CNTM_0068	1	CNTM_0068_1	93	GCF_000438845.1.report	94.26	99.24
CNTM_0069	1	CNTM_0069_1	84	GCF_000438845.1.report	94.27	99.24
CNTM_0070	1	CNTM_0070_1	94	GCF_000438845.1.report	94.31	99.26
CNTM_0067	1	CNTM_0067_1	128	GCF_000438845.1.report	80.27	91.5
CNTM_0071	1	CNTM_0071_1	125	GCF_000438845.1.report	94.92	99.3

CNTM_0148	1	CNTM_0148_3	102	GCF_000438845.1.report		94.95	99.29
CNTM_0149	1	CNTM_0149_3	106	GCF_000438845.1.report		94.96	99.31
CNTM_0150	1	CNTM_0150_3	187	GCF_000438845.1.report		89	98
CNTM_0072	1	CNTM_0072_4	227	GCF_012317185.1		81.34	97.43
CNTM_0073	1	CNTM_0073_1	60	GCF_000438845.1.report		91.07	99.15
CNTM_0074	1	CNTM_0074_1	113	GCF_000438845.1.report		91.05	99.15
CNTM_0075	1	CNTM_0075_1	67	GCF_000438845.1.report		95.92	99.34
CNTM_0076	1	CNTM_0076_1	105	GCF_000438845.1.report		93.09	98.81
CNTM_0077	1	CNTM_0077_1	108	GCF_000438845.1.report		94.69	99.31
CNTM_0078	1	CNTM_0078_1	97	GCF_000438845.1.report		91.31	99.11
CNTM_0079	1	CNTM_0079_1	85	GCF_000438845.1.report		95.55	99.35
CNTM_0080	1	CNTM_0080_1	84	GCF_000438845.1.report		94.58	99.34
CNTM_0081	1	CNTM_0081_1	92	GCF_000438845.1.report		95.21	99.3
CNTM_0083	1	CNTM_0083_1	96	GCF_000438845.1.report		96.06	99.35
CNTM_0082	1	CNTM_0082_1	322	GCF_000438845.1.report		96.14	99.39
CNTM_0084	1	CNTM_0084_1	85	GCF_000438845.1.report		96.07	99.36
CNTM_0085	1	CNTM_0085_1	84	GCF_000438845.1.report		96.08	99.35
CNTM_0086	0	0	#N/A		0	0	0
CNTM_0087	0	0	#N/A		0	0	0
CNTM_0088	1	CNTM_0088_1	100	GCF_000438845.1.report		96.06	99.36
CNTM_0089	1	CNTM_0089_1	91	GCF_000438845.1.report		96.07	99.35
CNTM_0090	1	CNTM_0090_1	133	GCF_000438845.1.report		86.57	99.34
CNTM_0091	1	CNTM_0091_1	74	GCF_000438845.1.report		96.08	99.37
CNTM_0092	1	CNTM_0092_1	97	GCF_000438845.1.report		93.04	99.31
CNTM_0093	1	CNTM_0093_1	260	GCF_000438845.1.report		94.59	99.28
CNTM_0094	1	CNTM_0094_1	173	GCF_000438845.1.report		94.54	99.25
CNTM_0095	1	CNTM_0095_1	158	GCF_000438845.1.report		93.32	99.22
CNTM_0096	1	CNTM_0096_1	132	GCF_000438845.1.report		89.09	98.04
CNTM_0097	1	CNTM_0097_1	88	GCF_000438845.1.report		94.33	99.25
CNTM_0098	1	CNTM_0098_1	78	GCF_000438845.1.report		95.83	99.37
CNTM_0099	1	CNTM_0099_1	131	GCF_000438845.1.report		91.5	99.1
CNTM_0100	1	CNTM_0100_1	150	GCF_000438845.1.report		91.51	99.1
CNTM_0101	1	CNTM_0101_1	119	GCF_000438845.1.report		91.72	99.11
CNTM_0102	1	CNTM_0102_1	126	GCF_000438845.1.report		92.45	99.16
CNTM_0104	1	CNTM_0104_1	127	GCF_000438845.1.report		92.13	98.76
CNTM_0105	1	CNTM_0105_1	128	GCF_000438845.1.report		92.13	98.77
CNTM_0106	1	CNTM_0106_1	102	GCF_000438845.1.report		94.22	99.34
CNTM_0107	1	CNTM_0107_1	63	GCF_000438845.1.report		94.26	99.32
CNTM_0108	1	CNTM_0108_1	86	GCF_000438845.1.report		90.91	99.2

CNTM_0109	1	CNTM_0109_1	69	GCF_000438845.1.report	96.07	99.36
CNTM_0110	1	CNTM_0110_1	85	GCF_000438845.1.report	91.24	99.32
CNTM_0111	1	CNTM_0111_1	88	GCF_000438845.1.report	95.37	99.38
CNTM_0112	1	CNTM_0112_1	129	GCF_000438845.1.report	94.64	99.28
CNTM_0114	1	CNTM_0114_1	233	GCF_000438845.1.report	94.67	99.26
CNTM_0113	1	CNTM_0113_1	267	GCF_000438845.1.report	94.67	99.27
CNTM_0115	1	CNTM_0115_1	735	GCF_000438845.1.report	89.15	99.13
CNTM_0116	1	CNTM_0116_1	90	GCF_000438845.1.report	94.62	99.25
CNTM_0151	1	CNTM_0151_4	50	GCF_000438845.1.report	94.21	99.27
CNTM_0120	1	CNTM_0120_1	63	GCF_000438845.1.report	94.35	99.27
CNTM_0117	1	CNTM_0117_1	63	GCF_000438845.1.report	94.06	99.27
CNTM_0118	1	CNTM_0118_1	68	GCF_000438845.1.report	94.22	99.28
CNTM_0152	1	CNTM_0152_3	373	GCF_000438845.1.report	94.2	99.3
CNTM_0153	1	CNTM_0153_3	200	GCF_000438845.1.report	94.19	99.28
CNTM_0119	1	CNTM_0119_1	81	GCF_000438845.1.report	94.22	99.28
CNTM_0121	1	CNTM_0121_1	74	GCF_000438845.1.report	95.2	99.28
CNTM_0154	1	CNTM_0154_3	127	GCF_000438845.1.report	95.21	99.28
CNTM_0123	1	CNTM_0123_1	85	GCF_000438845.1.report	93.99	99.3
CNTM_0155	1	CNTM_0155_3	97	GCF_000438845.1.report	93.98	99.3
CNTM_0122	1	CNTM_0122_1	84	GCF_000438845.1.report	94.01	99.3
CNTM_0124	1	CNTM_0124_1	87	GCF_000438845.1.report	93.98	99.3
CNTM_0125	1	CNTM_0125_1	138	GCF_000438845.1.report	94.57	99.24
CNTM_0126	1	CNTM_0126_1	115	GCF_000438845.1.report	93.01	99.25
CNTM_0127	1	CNTM_0127_1	80	GCF_000438845.1.report	95.32	99.35
CNTM_0156	1	CNTM_0156_3	97	GCF_000438845.1.report	95.33	99.35
CNTM_0128	1	CNTM_0128_1	87	GCF_000438845.1.report	94.98	99.3
CNTM_0129	1	CNTM_0129_1	98	GCF_000438845.1.report	93.23	96.37
CNTM_0157	1	CNTM_0157_3	92	GCF_000438845.1.report	94.19	99.27
CNTM_0130	1	CNTM_0130_1	122	GCF_000438845.1.report	96.23	99.33
CNTM_0131	0	0	#N/A	0	0	0
CNTM_0132	1	CNTM_0132_1	137	GCF_000438845.1.report	91.95	98.75
CNTM_0137	1	CNTM_0137_1	61	GCF_000438845.1.report	95.98	99.82
CNTM_0133	1	CNTM_0133_1	63	GCF_000438845.1.report	95.97	99.82
CNTM_0135	1	CNTM_0135_1	91	GCF_000438845.1.report	93.26	99.28
CNTM_0134	1	CNTM_0134_1	93	GCF_000438845.1.report	95.92	99.81
CNTM_0136	1	CNTM_0136_1	71	GCF_000438845.1.report	95.98	99.81
CNTM_0138	1	CNTM_0138_1	96	GCF_000438845.1.report	95.97	99.82
CNTM_0139	1	CNTM_0139_1	152	GCF_000438845.1.report	91.38	99.21
CNTM_0140	1	CNTM_0140_1	89	GCF_000438845.1.report	94.94	99.29

MASH Species	Source	Patient	Episode	Day
Clostridioides difficile ATCC 9689 = DSM 1296 (firmicutes)	stool	11	E1	133
Clostridioides difficile ATCC 9689 = DSM 1296 (firmicutes)	stool	14	E2	147
Clostridioides difficile ATCC 9689 = DSM 1296 (firmicutes)	bedrail	14	E2	148
Clostridioides difficile ATCC 9689 = DSM 1296 (firmicutes)	stool	21	E1	133
Clostridioides difficile ATCC 9689 = DSM 1296 (firmicutes)	stool	23	E1	133
Clostridioides difficile ATCC 9689 = DSM 1296 (firmicutes)	bedrail	23	E1	141
Clostridioides difficile ATCC 9689 = DSM 1296 (firmicutes)	stool	23	E1	141
Clostridioides difficile ATCC 9689 = DSM 1296 (firmicutes)	stool	25	E1	140
Clostridioides difficile ATCC 9689 = DSM 1296 (firmicutes)	stool	25	E1	143
Clostridioides difficile ATCC 9689 = DSM 1296 (firmicutes)	stool	25	E1	148
Clostridioides difficile ATCC 9689 = DSM 1296 (firmicutes)	bedrail	25	E1	148
Clostridioides difficile ATCC 9689 = DSM 1296 (firmicutes)	stool	44	E1	163
Clostridioides difficile ATCC 9689 = DSM 1296 (firmicutes)	bedrail	44	E1	165
0	bedrail	44	E1	165
Clostridioides difficile ATCC 9689 = DSM 1296 (firmicutes)	stool	47	E1	166
Clostridioides difficile ATCC 9689 = DSM 1296 (firmicutes)	stool	55	E1	189
Clostridioides difficile ATCC 9689 = DSM 1296 (firmicutes)	stool	65	E1	196
Clostridioides difficile ATCC 9689 = DSM 1296 (firmicutes)	stool	2001	E1	15
Clostridioides difficile ATCC 9689 = DSM 1296 (firmicutes)	stool	2002	E3	74
Clostridioides difficile ATCC 9689 = DSM 1296 (firmicutes)	bedrail	2003	E1	16
Clostridioides difficile ATCC 9689 = DSM 1296 (firmicutes)	rectal swab	2003	E1	16
Clostridioides difficile ATCC 9689 = DSM 1296 (firmicutes)	bedrail	2003	E1	21
Clostridioides difficile ATCC 9689 = DSM 1296 (firmicutes)	stool	2003	E1	21
Clostridioides difficile ATCC 9689 = DSM 1296 (firmicutes)	stool	2004	E1	14
Clostridioides difficile ATCC 9689 = DSM 1296 (firmicutes)	stool	2004	E1	21
Clostridioides difficile ATCC 9689 = DSM 1296 (firmicutes)	stool	2007	E1	15
Clostridioides difficile ATCC 9689 = DSM 1296 (firmicutes)	stool	2008	E1	16
Clostridioides difficile ATCC 9689 = DSM 1296 (firmicutes)	stool	2008	E1	21
Clostridioides difficile ATCC 9689 = DSM 1296 (firmicutes)	rectal swab	2008	E2	80
0	stool	2013	E1	17
Clostridioides difficile ATCC 9689 = DSM 1296 (firmicutes)	rectal swab	2015	E1	30
Clostridioides difficile ATCC 9689 = DSM 1296 (firmicutes)	stool	2018	E1	21
Genomospecies	stool	2019	E1	22
Genomospecies	stool	2019	E1	30
Clostridioides difficile ATCC 9689 = DSM 1296 (firmicutes)	stool	2024	E2	72
Clostridioides difficile ATCC 9689 = DSM 1296 (firmicutes)	stool	2024	E2	84
Clostridioides difficile ATCC 9689 = DSM 1296 (firmicutes)	stool	2026	E4	122
Clostridioides difficile ATCC 9689 = DSM 1296 (firmicutes)	stool	2026	E4	127

Clostridioides difficile ATCC 9689 = DSM 1296 (firmicutes)	bedrail	2026	E4	127
Clostridioides difficile ATCC 9689 = DSM 1296 (firmicutes)	stool	2026	E5	189
Clostridioides difficile ATCC 9689 = DSM 1296 (firmicutes)	bedrail	2027	E1	23
Genomospecies	stool	2030	E1	23
Genomospecies	stool	2030	E1	23
0	stool	2033	E1	37
Clostridioides difficile ATCC 9689 = DSM 1296 (firmicutes)	rectal swab	2035	E1	25
Clostridioides difficile ATCC 9689 = DSM 1296 (firmicutes)	stool	2038	E1	25
Clostridioides difficile ATCC 9689 = DSM 1296 (firmicutes)	stool	2043	E1	29
Clostridioides difficile ATCC 9689 = DSM 1296 (firmicutes)	stool	2044	E1	29
Clostridioides difficile ATCC 9689 = DSM 1296 (firmicutes)	rectal swab	2044	E1	35
Clostridioides difficile ATCC 9689 = DSM 1296 (firmicutes)	bedrail	2044	E1	42
Clostridioides difficile ATCC 9689 = DSM 1296 (firmicutes)	rectal swab	2044	E1	42
Clostridioides difficile ATCC 9689 = DSM 1296 (firmicutes)	bedrail	2044	E1	51
Clostridioides difficile ATCC 9689 = DSM 1296 (firmicutes)	rectal swab	2044	E1	51
Clostridioides difficile ATCC 9689 = DSM 1296 (firmicutes)	bedrail	2050	E1	30
Clostridioides difficile ATCC 9689 = DSM 1296 (firmicutes)	stool	2050	E1	30
Clostridioides difficile ATCC 9689 = DSM 1296 (firmicutes)	rectal swab	2051	E1	32
Clostridioides difficile ATCC 9689 = DSM 1296 (firmicutes)	rectal swab	2051	E1	44
Clostridioides difficile ATCC 9689 = DSM 1296 (firmicutes)	stool	2056	E1	31
Clostridioides difficile ATCC 9689 = DSM 1296 (firmicutes)	stool	2056	E1	34
Clostridioides difficile ATCC 9689 = DSM 1296 (firmicutes)	stool	2056	E1	36
Clostridioides difficile ATCC 9689 = DSM 1296 (firmicutes)	stool	2056	E3	72
Clostridioides difficile ATCC 9689 = DSM 1296 (firmicutes)	stool	2056	E3	77
Clostridioides difficile ATCC 9689 = DSM 1296 (firmicutes)	bedrail	2056	E3	78
Clostridioides difficile ATCC 9689 = DSM 1296 (firmicutes)	stool	2056	E3	78
Clostridioides difficile ATCC 9689 = DSM 1296 (firmicutes)	rectal swab	2061	E1	59
Clostridioides difficile ATCC 9689 = DSM 1296 (firmicutes)	stool	2063	E1	42
Clostridioides difficile ATCC 9689 = DSM 1296 (firmicutes)	stool	2068	E3	139
Clostridioides difficile ATCC 9689 = DSM 1296 (firmicutes)	stool	2070	E2	118
Clostridioides difficile ATCC 9689 = DSM 1296 (firmicutes)	stool	2070	E2	120
Clostridioides difficile ATCC 9689 = DSM 1296 (firmicutes)	stool	2070	E2	122
Clostridioides difficile ATCC 9689 = DSM 1296 (firmicutes)	rectal swab	2088	E1	49
Clostridioides difficile ATCC 9689 = DSM 1296 (firmicutes)	rectal swab	2088	E1	58
Clostridioides difficile ATCC 9689 = DSM 1296 (firmicutes)	stool	2088	E1	64
Genomospecies	keyboard	2088	E1	64
Clostridioides difficile ATCC 9689 = DSM 1296 (firmicutes)	stool	2093	E1	58
Clostridioides difficile ATCC 9689 = DSM 1296 (firmicutes)	bedrail	2093	E1	58
Clostridioides difficile ATCC 9689 = DSM 1296 (firmicutes)	stool	2093	E1	59

Clostridioides difficile ATCC 9689 = DSM 1296 (firmicutes)	keyboard	2093	E1	63
[Clostridium] innocuum (firmicutes)	stool	2094	E1	50
Clostridioides difficile ATCC 9689 = DSM 1296 (firmicutes)	stool	2094	E2	70
Clostridioides difficile ATCC 9689 = DSM 1296 (firmicutes)	stool	2094	E3	132
Clostridioides difficile ATCC 9689 = DSM 1296 (firmicutes)	rectal swab	2095	E1	52
Clostridioides difficile ATCC 9689 = DSM 1296 (firmicutes)	rectal swab	2095	E3	81
Clostridioides difficile ATCC 9689 = DSM 1296 (firmicutes)	stool	2100	E1	59
Clostridioides difficile ATCC 9689 = DSM 1296 (firmicutes)	stool	2104	E2	124
Clostridioides difficile ATCC 9689 = DSM 1296 (firmicutes)	bedrail	2106	E2	155
Clostridioides difficile ATCC 9689 = DSM 1296 (firmicutes)	stool	2106	E2	155
Clostridioides difficile ATCC 9689 = DSM 1296 (firmicutes)	stool	2107	E1	57
Clostridioides difficile ATCC 9689 = DSM 1296 (firmicutes)	stool	2109	E2	85
Clostridioides difficile ATCC 9689 = DSM 1296 (firmicutes)	bedrail	2109	E2	91
Clostridioides difficile ATCC 9689 = DSM 1296 (firmicutes)	stool	2109	E2	91
Clostridioides difficile ATCC 9689 = DSM 1296 (firmicutes)	stool	2109	E2	98
0	stool	2109	E2	106
0	stool	2109	E2	112
Clostridioides difficile ATCC 9689 = DSM 1296 (firmicutes)	stool	2109	E2	120
Clostridioides difficile ATCC 9689 = DSM 1296 (firmicutes)	stool	2109	E2	133
Clostridioides difficile ATCC 9689 = DSM 1296 (firmicutes)	stool	2109	E2	150
Clostridioides difficile ATCC 9689 = DSM 1296 (firmicutes)	stool	2109	E2	165
Clostridioides difficile ATCC 9689 = DSM 1296 (firmicutes)	stool	2111	E2	69
Clostridioides difficile ATCC 9689 = DSM 1296 (firmicutes)	stool	2132	E1	67
Clostridioides difficile ATCC 9689 = DSM 1296 (firmicutes)	stool	2132	E2	93
Clostridioides difficile ATCC 9689 = DSM 1296 (firmicutes)	rectal swab	2135	E1	80
Clostridioides difficile ATCC 9689 = DSM 1296 (firmicutes)	rectal swab	2142	E1	77
Clostridioides difficile ATCC 9689 = DSM 1296 (firmicutes)	stool	2163	E1	144
Clostridioides difficile ATCC 9689 = DSM 1296 (firmicutes)	bedrail	2167	E1	98
Clostridioides difficile ATCC 9689 = DSM 1296 (firmicutes)	bedrail	2167	E1	105
Clostridioides difficile ATCC 9689 = DSM 1296 (firmicutes)	bedrail	2167	E1	119
Clostridioides difficile ATCC 9689 = DSM 1296 (firmicutes)	stool	2178	E1	93
Clostridioides difficile ATCC 9689 = DSM 1296 (firmicutes)	stool	2187	E1	106
Clostridioides difficile ATCC 9689 = DSM 1296 (firmicutes)	stool	2191	E1	112
Clostridioides difficile ATCC 9689 = DSM 1296 (firmicutes)	stool	2191	E1	112
Clostridioides difficile ATCC 9689 = DSM 1296 (firmicutes)	stool	2195	E1	101
Clostridioides difficile ATCC 9689 = DSM 1296 (firmicutes)	stool	2195	E1	111
Clostridioides difficile ATCC 9689 = DSM 1296 (firmicutes)	stool	2198	E1	105
Clostridioides difficile ATCC 9689 = DSM 1296 (firmicutes)	stool	2203	E2	176
Clostridioides difficile ATCC 9689 = DSM 1296 (firmicutes)	stool	2205	E1	110

Clostridioides difficile ATCC 9689 = DSM 1296 (firmicutes)	stool	2230	E1	126
Clostridioides difficile ATCC 9689 = DSM 1296 (firmicutes)	bedrail	2238	E1	124
Clostridioides difficile ATCC 9689 = DSM 1296 (firmicutes)	stool	2238	E1	124
Clostridioides difficile ATCC 9689 = DSM 1296 (firmicutes)	bedrail	2238	E1	134
Clostridioides difficile ATCC 9689 = DSM 1296 (firmicutes)	stool	2238	E1	134
Clostridioides difficile ATCC 9689 = DSM 1296 (firmicutes)	stool	2238	E1	135
Clostridioides difficile ATCC 9689 = DSM 1296 (firmicutes)	stool	2245	E1	142
Clostridioides difficile ATCC 9689 = DSM 1296 (firmicutes)	stool	2245	E1	150
Clostridioides difficile ATCC 9689 = DSM 1296 (firmicutes)	stool	2245	E1	151
Clostridioides difficile ATCC 9689 = DSM 1296 (firmicutes)	stool	2245	E1	156
Clostridioides difficile ATCC 9689 = DSM 1296 (firmicutes)	stool	2245	E2	182
Clostridioides difficile ATCC 9689 = DSM 1296 (firmicutes)	stool	2245	E2	189
Clostridioides difficile ATCC 9689 = DSM 1296 (firmicutes)	rectal swab	2245	E2	200
Clostridioides difficile ATCC 9689 = DSM 1296 (firmicutes)	stool	2262	E1	146
Clostridioides difficile ATCC 9689 = DSM 1296 (firmicutes)	stool	2262	E1	150
Clostridioides difficile ATCC 9689 = DSM 1296 (firmicutes)	stool	2266	E1	140
Clostridioides difficile ATCC 9689 = DSM 1296 (firmicutes)	stool	2266	E1	148
Clostridioides difficile ATCC 9689 = DSM 1296 (firmicutes)	stool	2266	E1	154
Clostridioides difficile ATCC 9689 = DSM 1296 (firmicutes)	stool	2266	E1	156
Clostridioides difficile ATCC 9689 = DSM 1296 (firmicutes)	rectal swab	2273	E1	144
Clostridioides difficile ATCC 9689 = DSM 1296 (firmicutes)	stool	2278	E1	145
Clostridioides difficile ATCC 9689 = DSM 1296 (firmicutes)	stool	2285	E1	154
Clostridioides difficile ATCC 9689 = DSM 1296 (firmicutes)	stool	2285	E1	158
Clostridioides difficile ATCC 9689 = DSM 1296 (firmicutes)	stool	2292	E1	155
Clostridioides difficile ATCC 9689 = DSM 1296 (firmicutes)	stool	2293	E1	168
Clostridioides difficile ATCC 9689 = DSM 1296 (firmicutes)	keyboard	2293	E1	168
Clostridioides difficile ATCC 9689 = DSM 1296 (firmicutes)	bedrail	2298	E1	162
0	stool	2298	E1	162
Clostridioides difficile ATCC 9689 = DSM 1296 (firmicutes)	stool	2302	E1	166
Clostridioides difficile ATCC 9689 = DSM 1296 (firmicutes)	stool	2330	E1	183
Clostridioides difficile ATCC 9689 = DSM 1296 (firmicutes)	bedrail	2330	E1	189
Clostridioides difficile ATCC 9689 = DSM 1296 (firmicutes)	stool	2330	E1	189
Clostridioides difficile ATCC 9689 = DSM 1296 (firmicutes)	keyboard	2330	E1	196
Clostridioides difficile ATCC 9689 = DSM 1296 (firmicutes)	stool	2330	E1	196
Clostridioides difficile ATCC 9689 = DSM 1296 (firmicutes)	stool	2336	E1	189
Clostridioides difficile ATCC 9689 = DSM 1296 (firmicutes)	rectal swab	2341	E1	196
Clostridioides difficile ATCC 9689 = DSM 1296 (firmicutes)	stool	2342	E1	194

Room	MLST	Clade	NAAT	EIA_test	EIA_result
A_04	125	1	ND	0	NA

B_12	6	1	1	1	1
B_12	6	1	NA	0	NA
A_01	53	1	1	1	0
A_02	10	1	1	0	NA
A_02	10	1	NA	0	NA
A_02	10	1	1	0	NA
A_03	6	1	ND		
A_03	6	1	1	1	0
A_03	6	1	ND	0	NA
A_03	6	1	NA		
A_07	11	5	1	1	1
A_07	11	5	NA	0	NA
A_07	NA	#N/A	NA		
A_05	42	1	0	0	NA
A_03	8	1	ND	0	NA
A_01	83	1	0	0	NA
B_21	2	1	1	0	NA
B_27	12	1	1	0	NA
B_06	83	1	NA	0	NA
B_06	83	1	ND	0	NA
B_12	83	1	NA	0	NA
B_12	83	1	0	0	NA
B_02	2	1	1	0	NA
B_02	2	1	1	0	NA
B_16	26	1	0	0	NA
B_24	9	1	0	0	NA
B_22	9	1	0	0	NA
B_14	42	1	ND	0	NA
B_04	NA	#N/A	1	0	NA
B_03	2	1	ND	0	NA
B_20	2	1	ND	0	NA
B_29	763	C1	0	0	NA
B_29	763	C1	0	0	NA
B_09	42	1	1	0	NA
B_09	42	1	1	0	NA
B_02	110	1	1	1	0
B_02	110	1	ND	0	NA
B_02	110	1	NA	0	NA
B_06	110	1	1	1	1

B_24	53	1	NA	0	NA
B_11	-	C1	0	0	NA
B_11	-	C1	0	1	0
B_31	NA	#N/A	1	0	NA
B_16	15	1	0	0	NA
B_26	2	1	1	0	NA
B_20	34	1	1	0	NA
B_06	15	1	0	0	NA
B_06	15	1	0	0	NA
B_06	53	1	NA	0	NA
B_06	15	1	0	0	NA
B_06	53	1	NA	0	NA
B_06	15	1	0	0	NA
B_24	53	1	NA	0	NA
B_24	42	1	1	0	NA
B_25	49	1	1	0	NA
B_25	49	1	1	0	NA
B_04	53	1	1	0	NA
B_04	53	1	1	1	1
B_04	53	1	1	0	NA
B_03	53	1	ND	0	NA
B_04	53	1	1	1	1
B_04	53	1	NA	0	NA
B_04	53	1	1	0	NA
B_21	54	1	ND	0	NA
B_29	35	1	ND	0	NA
B_12	39	4	0	0	NA
A_06	110	1	1	1	0
A_06	110	1	ND		
A_06	110	1	1	1	0
B_30	15	1	0	0	NA
B_30	15	1	0	0	NA
B_30	15	1	0	0	NA
B_30	-	C1	NA	0	NA
B_02	42	1	1	1	0
B_02	42	1	NA		
B_02	42	1	ND		
B_02	37	4	NA		
B_13	NA	#N/A	0		

B_24	26	1	ND		
B_26	26	1	0	1	0
B_11	42	1	0		
B_31	1	2	0		
B_28	14	1	1	1	1
B_01	26	1	0		
B_17	53	1	NA		
B_17	8	1	1		
B_23	2	1	1		
B_06	42	1	1		
B_06	42	1	NA		
B_06	42	1	1		
B_06	42	1	1		
B_06	NA	#N/A	1		
B_06	NA	#N/A	1		
B_06	42	1	0		
B_06	42	1	0		
B_06	42	1	1		
B_06	42	1	1		
B_25	43	1	1	1	1
B_20	103	1	1		
B_09	-	1	1		
B_19	2	1	ND		
B_20	-	4	0		
B_04	53	1	1	1	1
B_25	8	1	NA		
B_25	26	1	NA		
B_25	26	1	NA		
B_05	54	1	1		
B_07	34	1	1	1	0
B_18	41	2	1		
B_18	41	2	1	1	1
B_08	8	1	1		
B_08	8	1	0	1	0
B_27	3	1	ND		
B_12	42	1	1		
B_16	6	1	0	1	0
B_04	8	1	ND		
B_13	2	1	NA		

B_13	2	1	0		
B_13	2	1	NA		
B_13	2	1	1		
B_13	2	1	1	1	0
B_20	15	1	ND		
B_20	15	1	0	1	1
B_20	15	1	0		
B_20	15	1	0		
B_19	15	1	ND		
B_19	15	1	ND		
B_19	15	1	ND		
B_15	2	1	1	1	0
B_15	2	1	ND		
B_10	14	1	1	1	0
B_10	14	1	ND		
B_10	14	1	ND		
B_10	14	1	0	1	0
B_09	34	1	1		
B_08	15	1	0		
B_04	21	1	1	1	0
B_04	21	1	ND		
B_23	2	1	1		
B_28	11	5	ND		
B_28	15	1	NA		
B_25	53	1	NA		
B_25	NA	#N/A	1		
B_12	41	2	1		
B_16	3	1	1	1	0
B_16	3	1	NA		
B_16	83	1	1		
B_16	3	1	NA		
B_16	3	1	1		
B_22	3	1	0		
B_12	83	1	0		
B_11	2	1	1		
TcdA	TcdB	CdtR	CdtAB	source2	mlst_network
0	0	0	0	carrier	None
1	1	1	pseudo	CDI	45
1	1	1	pseudo	bedrail	45

1	1	1	pseudo	carrier	None
1	1	1	pseudo	carrier	1
1	1	1	pseudo	bedrail	1
1	1	1	pseudo	carrier	1
1	1	1	pseudo	carrier	46
1	1	1	pseudo	carrier	46
1	1	1	pseudo	carrier	46
1	1	1	pseudo	bedrail	46
1	1	1	1	CDI	4
1	1	1	1	bedrail	4
#N/A	#N/A	#N/A	#N/A	bedrail	None
1	1	1	pseudo	carrier	None
1	1	1	pseudo	carrier	None
0	0	0	0	carrier	None
1	1	1	pseudo	carrier	17
1	1	1	pseudo	carrier	None
0	0	0	0	bedrail	50
0	0	0	0	carrier	50
0	0	0	0	bedrail	50
0	0	0	0	carrier	50
1	1	1	pseudo	carrier	18
1	1	1	pseudo	carrier	18
0	0	0	0	carrier	None
0	0	0	0	carrier	None
0	0	0	0	carrier	None
1	1	1	pseudo	carrier	None
#N/A	#N/A	#N/A	#N/A	carrier	None
1	1	1	pseudo	carrier	None
1	1	1	pseudo	carrier	17
0	0	0	1	carrier	48
0	0	0	1	carrier	48
1	1	0	pseudo	carrier	35
1	1	1	pseudo	carrier	35
1	1	1	pseudo	carrier	2
1	1	1	pseudo	carrier	2
1	1	1	pseudo	bedrail	2
1	1	1	pseudo	CDI	2
1	1	1	pseudo	bedrail	42
0	0	0	0	carrier	None

0	0	0	0	carrier	None
#N/A	#N/A	#N/A	#N/A	carrier	None
0	0	0	0	carrier	None
1	1	1	pseudo	carrier	None
1	1	1	pseudo	carrier	None
0	0	0	0	carrier	8
0	0	0	0	carrier	8
1	1	1	pseudo	bedrail	42
0	0	0	0	carrier	8
1	1	1	pseudo	bedrail	42
0	0	0	0	carrier	8
1	1	1	pseudo	bedrail	42
1	1	1	pseudo	carrier	None
1	1	1	pseudo	carrier	None
1	1	1	pseudo	carrier	None
1	1	1	pseudo	carrier	42
1	1	1	pseudo	CDI	42
1	1	1	pseudo	CDI	42
1	1	1	pseudo	CDI	42
1	1	1	pseudo	CDI	42
1	1	1	pseudo	bedrail	42
1	1	1	pseudo	CDI	42
1	1	1	pseudo	carrier	None
1	1	0	0	carrier	None
0	0	0	0	carrier	None
1	1	1	pseudo	carrier	3
1	1	1	pseudo	carrier	3
1	1	1	pseudo	carrier	3
0	0	0	0	carrier	9
0	0	0	0	carrier	9
0	0	0	0	carrier	9
0	0	0	1	keyboard	None
1	1	1	pseudo	carrier	37
1	1	1	pseudo	bedrail	37
1	1	1	pseudo	carrier	37
1	1	0	0	keyboard	None
#N/A	#N/A	#N/A	#N/A	carrier	None
0	0	0	0	carrier	14
0	0	0	0	carrier	14

1	1	1	pseudo	carrier	None
1	1	1	1	carrier	None
1	1	1	pseudo	CDI	None
0	0	0	0	carrier	None
1	1	1	pseudo	bedrail	42
1	1	1	pseudo	carrier	None
1	1	1	pseudo	carrier	None
1	1	1	pseudo	carrier	39
1	1	1	pseudo	bedrail	39
1	1	1	pseudo	carrier	39
1	1	1	pseudo	carrier	39
#N/A	#N/A	#N/A	#N/A	carrier	None
#N/A	#N/A	#N/A	#N/A	carrier	None
1	1	1	pseudo	carrier	39
1	1	1	pseudo	carrier	39
1	1	1	pseudo	carrier	39
1	1	1	pseudo	carrier	39
1	1	1	pseudo	CDI	None
1	1	1	pseudo	carrier	None
1	1	1	pseudo	carrier	None
1	1	1	pseudo	carrier	None
0	0	0	0	carrier	None
1	1	1	pseudo	CDI	None
1	1	1	pseudo	bedrail	None
0	0	0	0	bedrail	16
0	0	0	0	bedrail	16
1	1	1	pseudo	carrier	None
1	1	1	pseudo	carrier	None
1	1	1	1	CDI	32
1	1	1	1	CDI	32
1	1	1	pseudo	carrier	55
1	1	1	pseudo	carrier	55
0	0	0	0	carrier	None
1	1	1	pseudo	carrier	None
1	1	1	pseudo	carrier	None
1	1	1	pseudo	carrier	None
1	1	1	pseudo	bedrail	24
1	1	1	pseudo	carrier	24
1	1	1	pseudo	bedrail	24

1	1	1	pseudo	carrier	24
1	1	1	pseudo	carrier	24
0	0	0	0	CDI	10
0	0	0	0	CDI	10
0	0	0	0	CDI	10
0	0	0	0	CDI	10
0	0	0	0	carrier	10
0	0	0	0	carrier	10
0	0	0	0	carrier	10
1	1	1	pseudo	carrier	None
1	1	1	pseudo	carrier	None
1	1	1	pseudo	carrier	6
1	1	1	pseudo	carrier	6
1	1	1	pseudo	carrier	6
1	1	1	pseudo	carrier	6
1	1	1	pseudo	carrier	None
0	0	0	0	carrier	None
1	1	1	pseudo	carrier	12
1	1	1	pseudo	carrier	12
1	1	1	pseudo	carrier	None
1	1	1	1	carrier	None
0	0	0	0	keyboard	10
1	1	1	pseudo	bedrail	None
#N/A	#N/A	#N/A	#N/A	carrier	None
1	1	1	1	carrier	None
1	1	1	pseudo	carrier	31
1	1	1	pseudo	bedrail	31
0	0	0	0	carrier	None
1	1	1	pseudo	keyboard	31
1	1	1	pseudo	carrier	31
1	1	1	pseudo	carrier	31
0	0	0	0	carrier	None
1	1	1	pseudo	carrier	None

Supplementary Table 2: Figure 2c references.

Reference accession number	ST	Clade
ERR3284197	2	1
ERR1853149	8	1
SRR1801764	3	1
SRR3631351	1	2
SRR2915782	41	2
SRR2915710	67	2
SRR3630588	5	3
SRR6008966	22	3
SRR6986097	221	3
SRR2915746	37	4
ERR272064	39	4
SRR1735384	81	4
SRR6009820	11	5
SRR3631169	167	5
SRR3631291	258	5
ERR232399	178	C1
SRR3654510	177	C1
ERR789085	641	C1
SRR3654506	200	C2
SRR3629287	311	C2
ERR3296451	637	C2
ERR2216003	343	C3
ERR2215981	369	C3

Supplementary Table 3: Figure 4c references.

Reference
CDFMT-SF110_assembly_filtered
CDFMT-SF115_assembly_filtered
CDFMT-SF123_assembly_filtered
CNTM_0001_1
CNTM_0002_1
CNTM_0004_1
CNTM_0006_1
CNTM_0009_1
CNTM_0145_3
CNTM_0011_1
CNTM_0012_1
CNTM_0013_1
CNTM_0015_1
CNTM_0016_1
CNTM_0019_1
CNTM_0021_1
CNTM_0023_1
CNTM_0024_1
CNTM_0026_1
CNTM_0028_1
CNTM_0029_1
CNTM_0032_1
CNTM_0037_1
CNTM_0042_3
CNTM_0043_1
CNTM_0044_1
CNTM_0047_1
CNTM_0052_1
CNTM_0053_1
CNTM_0060_1
CNTM_0062_1
CNTM_0063_1
CNTM_0064_1
CNTM_0065_1
CNTM_0068_1
CNTM_0071_1
CNTM_0074_1
CNTM_0075_1
CNTM_0076_1
CNTM_0077_1
CNTM_0078_1
CNTM_0080_1
CNTM_0081_1
CNTM_0084_1
CNTM_0092_1
CNTM_0093_1
CNTM_0095_1
CNTM_0097_1
CNTM_0101_1
CNTM_0102_1
CNTM_0105_1
CNTM_0107_1
CNTM_0108_1
CNTM_0111_1

CNTM_0116_1
CNTM_0114_1
CNTM_0120_1
CNTM_0121_1
CNTM_0154_3
CNTM_0123_1
CNTM_0125_1
CNTM_0126_1
CNTM_0127_1
CNTM_0156_3
CNTM_0128_1
CNTM_0157_3
CNTM_0132_1
CNTM_0137_1
CNTM_0135_1
CNTM_0140_1
GCF_000003215.1_ASM321v1_genomic
GCF_000009205.2_ASM920v2_genomic
GCF_000027105.1_ASM2710v1_genomic
GCF_000085225.1_ASM8522v1_genomic
GCF_000154625.1_ASM15462v1_genomic
GCF_000154645.1_ASM15464v1_genomic
GCF_000154665.1_ASM15466v1_genomic
GCF_000154685.1_cdqaf_genomic
GCF_000155025.1_cduab_genomic
GCF_000155045.1_cdeaa_genomic
GCF_000155065.1_ASM15506v1_genomic
GCF_000164175.1_ASM16417v1_genomic
GCF_000164655.1_ASM16465v1_genomic
GCF_000210395.1_ASM21039v1_genomic
GCF_000210415.1_ASM21041v1_genomic
GCF_000210435.1_ASM21043v1_genomic
GCF_000210455.1_ASM21045v1_genomic
GCF_000211235.1_ASM21123v1_genomic
GCF_000235825.1_ASM23582v1_genomic
GCF_000235925.1_ASM23592v1_genomic
GCF_000270485.1_ASM27048v1_genomic
GCF_000376285.1_ASM37628v1_genomic
GCF_000438845.1_ASM43884v1_genomic
GCF_000448765.2_ASM44876v2_genomic
GCF_000448865.2_ASM44886v2_genomic
GCF_000448885.2_ASM44888v2_genomic
GCF_000448905.2_ASM44890v2_genomic
GCF_000448965.2_ASM44896v2_genomic
GCF_000448985.2_ASM44898v2_genomic
GCF_000449085.2_ASM44908v2_genomic
GCF_000449185.2_ASM44918v2_genomic
GCF_000449205.2_ASM44920v2_genomic
GCF_000449365.2_ASM44936v2_genomic
GCF_000449385.2_ASM44938v2_genomic
GCF_000449445.2_ASM44944v2_genomic
GCF_000449465.2_ASM44946v2_genomic
GCF_000449485.2_ASM44948v2_genomic
GCF_000449565.2_ASM44956v2_genomic
GCF_000449585.2_ASM44958v2_genomic
GCF_000449625.2_ASM44962v2_genomic
GCF_000449665.2_ASM44966v2_genomic

GCF_000449685.2_ASM44968v2_genomic
GCF_000449725.2_ASM44972v2_genomic
GCF_000449985.2_ASM44998v2_genomic
GCF_000450005.2_ASM45000v2_genomic
GCF_000450025.2_ASM45002v2_genomic
GCF_000450065.2_ASM45006v2_genomic
GCF_000450085.2_ASM45008v2_genomic
GCF_000450105.2_ASM45010v2_genomic
GCF_000450165.2_ASM45016v2_genomic
GCF_000450205.2_ASM45020v2_genomic
GCF_000450305.2_ASM45030v2_genomic
GCF_000450325.2_ASM45032v2_genomic
GCF_000450345.2_ASM45034v2_genomic
GCF_000450365.2_ASM45036v2_genomic
GCF_000450385.2_ASM45038v2_genomic
GCF_000450405.2_ASM45040v2_genomic
GCF_000450445.2_ASM45044v2_genomic
GCF_000450465.2_ASM45046v2_genomic
GCF_000450545.2_ASM45054v2_genomic
GCF_000450565.2_ASM45056v2_genomic
GCF_000450605.2_ASM45060v2_genomic
GCF_000450645.2_ASM45064v2_genomic
GCF_000450725.2_ASM45072v2_genomic
GCF_000450785.2_ASM45078v2_genomic
GCF_000450825.2_ASM45082v2_genomic
GCF_000450865.2_ASM45086v2_genomic
GCF_000451065.2_ASM45106v2_genomic
GCF_000451085.2_ASM45108v2_genomic
GCF_000451105.2_ASM45110v2_genomic
GCF_000451145.2_ASM45114v2_genomic
GCF_000451245.2_ASM45124v2_genomic
GCF_000451265.2_ASM45126v2_genomic
GCF_000451385.2_ASM45138v2_genomic
GCF_000451425.2_ASM45142v2_genomic
GCF_000451505.2_ASM45150v2_genomic
GCF_000451525.2_ASM45152v2_genomic
GCF_000451545.2_ASM45154v2_genomic
GCF_000451665.2_ASM45166v2_genomic
GCF_000451685.2_ASM45168v2_genomic
GCF_000451705.1_ASM45170v1_genomic
GCF_000451725.2_ASM45172v2_genomic
GCF_000451745.2_ASM45174v2_genomic
GCF_000451765.1_ASM45176v1_genomic
GCF_000451785.2_ASM45178v2_genomic
GCF_000451805.1_ASM45180v1_genomic
GCF_000451825.1_ASM45182v1_genomic
GCF_000451845.2_ASM45184v2_genomic
GCF_000451865.2_ASM45186v2_genomic
GCF_000451885.2_ASM45188v2_genomic
GCF_000451905.2_ASM45190v2_genomic
GCF_000451925.2_ASM45192v2_genomic
GCF_000451945.2_ASM45194v2_genomic
GCF_000451965.2_ASM45196v2_genomic
GCF_000451985.1_ASM45198v1_genomic
GCF_000452005.1_ASM45200v1_genomic
GCF_000452025.2_ASM45202v2_genomic
GCF_000452045.2_ASM45204v2_genomic

GCF_000452065.2_ASM45206v2_genomic
GCF_000452085.2_ASM45208v2_genomic
GCF_000452105.2_ASM45210v2_genomic
GCF_000452125.2_ASM45212v2_genomic
GCF_000452145.1_ASM45214v1_genomic
GCF_000452165.2_ASM45216v2_genomic
GCF_000452185.2_ASM45218v2_genomic
GCF_000452205.2_ASM45220v2_genomic
GCF_000452265.2_ASM45226v2_genomic
GCF_000452285.2_ASM45228v2_genomic
GCF_000452305.2_ASM45230v2_genomic
GCF_000452325.2_ASM45232v2_genomic
GCF_000452345.2_ASM45234v2_genomic
GCF_000473585.2_ASM47358v2_genomic
GCF_000473605.2_ASM47360v2_genomic
GCF_000473625.2_ASM47362v2_genomic
GCF_000473645.2_ASM47364v2_genomic
GCF_000473665.2_ASM47366v2_genomic
GCF_000473685.2_ASM47368v2_genomic
GCF_000473705.2_ASM47370v2_genomic
GCF_000530325.1_T42_genomic
GCF_000531315.1_E24_genomic
GCF_000531625.1_CD002_genomic
GCF_000531715.1_T61_genomic
GCF_000586575.1_WHS13_S12_genomic
GCF_000826625.1_G46R2_Q2_genomic
GCF_000828225.1_ASM82822v1_genomic
GCF_000828235.1_ASM82823v1_genomic
GCF_000828245.1_ASM82824v1_genomic
GCF_000828255.1_ASM82825v1_genomic
GCF_000828325.1_ASM82832v1_genomic
GCF_000932055.2_ASM93205v2_genomic
GCF_000949855.1_ASM94985v1_genomic
GCF_000953275.1_CD630DERM_genomic
GCF_001077535.1_ASM107753v2_genomic
GCF_001484885.1_CD26a54Rv1.0_genomic
GCF_001484895.1_CD26A54SV1.0_genomic
GCF_001577795.1_ASM157779v1_genomic
GCF_001678145.1_ASM167814v1_genomic
GCF_001757585.1_ASM175758v1_genomic
GCF_001757605.1_ASM175760v1_genomic
GCF_001757615.1_ASM175761v1_genomic
GCF_001757625.1_ASM175762v1_genomic
GCF_001857645.1_ASM185764v1_genomic
GCF_001971825.1_ASM197182v1_genomic
GCF_001971835.1_ASM197183v1_genomic
GCF_001971865.1_ASM197186v1_genomic
GCF_001971875.1_ASM197187v1_genomic
GCF_001971905.1_ASM197190v1_genomic
GCF_001971925.1_ASM197192v1_genomic
GCF_001971935.1_ASM197193v1_genomic
GCF_001971945.1_ASM197194v1_genomic
GCF_001971985.1_ASM197198v1_genomic
GCF_001972005.1_ASM197200v1_genomic
GCF_001972015.1_ASM197201v1_genomic
GCF_001972045.1_ASM197204v1_genomic
GCF_001972065.1_ASM197206v1_genomic

GCF_001972085.1_ASM197208v1_genomic
GCF_001972105.1_ASM197210v1_genomic
GCF_001972115.1_ASM197211v1_genomic
GCF_001972135.1_ASM197213v1_genomic
GCF_001972165.1_ASM197216v1_genomic
GCF_001972185.1_ASM197218v1_genomic
GCF_001972195.1_ASM197219v1_genomic
GCF_002007885.1_ASM200788v1_genomic
GCF_002073735.2_ASM207373v2_genomic
GCF_002080065.1_ASM208006v1_genomic
GCF_002082945.2_ASM208294v2_genomic
GCF_002082985.1_ASM208298v2_genomic
GCF_002165155.1_ASM216515v1_genomic
GCF_002234355.1_ASM223435v1_genomic
GCF_002300845.1_ASM230084v1_genomic
GCF_002300915.1_ASM230091v1_genomic
GCF_002300955.1_ASM230095v1_genomic
GCF_002301035.1_ASM230103v1_genomic
GCF_002301085.1_ASM230108v1_genomic
GCF_002301095.1_ASM230109v1_genomic
GCF_002301105.1_ASM230110v1_genomic
GCF_002301125.1_ASM230112v1_genomic
GCF_002301145.1_ASM230114v1_genomic
GCF_002301185.1_ASM230118v1_genomic
GCF_002301405.1_ASM230140v1_genomic
GCF_002301525.1_ASM230152v1_genomic
GCF_002301555.1_ASM230155v1_genomic
GCF_002301675.1_ASM230167v1_genomic
GCF_002301805.1_ASM230180v1_genomic
GCF_002301925.1_ASM230192v1_genomic
GCF_002302005.1_ASM230200v1_genomic
GCF_002302055.1_ASM230205v1_genomic
GCF_002302075.1_ASM230207v1_genomic
GCF_002302165.1_ASM230216v1_genomic
GCF_002302285.1_ASM230228v1_genomic
GCF_002302805.1_ASM230280v1_genomic
GCF_002303325.1_ASM230332v1_genomic
GCF_002303345.1_ASM230334v1_genomic
GCF_002303585.1_ASM230358v1_genomic
GCF_002304265.1_ASM230426v1_genomic
GCF_002304575.1_ASM230457v1_genomic
GCF_002304585.1_ASM230458v1_genomic
GCF_002335485.1_ASM233548v1_genomic
GCF_002352505.1_K40_RF17.1_genomic
GCF_002412385.1_K40_5754.1_genomic
GCF_002812585.1_ASM281258v1_genomic
GCF_002812605.1_ASM281260v1_genomic
GCF_002812625.1_ASM281262v1_genomic
GCF_002812645.1_ASM281264v1_genomic
GCF_002891495.1_ASM289149v1_genomic
GCF_002891605.1_ASM289160v1_genomic
GCF_002891615.1_ASM289161v1_genomic
GCF_002891625.1_ASM289162v1_genomic
GCF_002945415.1_ASM294541v1_genomic
GCF_002945515.1_ASM294551v1_genomic
GCF_002945665.1_ASM294566v1_genomic
GCF_002945755.1_ASM294575v1_genomic

GCF_002945855.1_ASM294585v1_genomic
GCF_002945945.1_ASM294594v1_genomic
GCF_002946035.1_ASM294603v1_genomic
GCF_002946135.1_ASM294613v1_genomic
GCF_002946195.1_ASM294619v1_genomic
GCF_002946515.2_ASM294651v2_genomic
GCF_002946535.2_ASM294653v2_genomic
GCF_002946555.2_ASM294655v2_genomic
GCF_002954015.1_ASM295401v1_genomic
GCF_002954285.1_ASM295428v1_genomic
GCF_002954305.1_ASM295430v1_genomic
GCF_003095675.1_ASM309567v1_genomic
GCF_003095695.1_ASM309569v1_genomic
GCF_003313545.1_ASM331354v1_genomic
GCF_003313565.1_ASM331356v1_genomic
GCF_003313585.1_ASM331358v1_genomic
GCF_003324015.1_ASM332401v1_genomic
GCF_003324025.1_ASM332402v1_genomic
GCF_003324035.1_ASM332403v1_genomic
GCF_003326835.1_ASM332683v1_genomic
GCF_003326845.1_ASM332684v1_genomic
GCF_003326875.1_ASM332687v1_genomic
GCF_003326885.1_ASM332688v1_genomic
GCF_003326915.1_ASM332691v1_genomic
GCF_003326935.1_ASM332693v1_genomic
GCF_003326965.1_ASM332696v1_genomic
GCF_003326985.1_ASM332698v1_genomic
GCF_003327015.1_ASM332701v1_genomic
GCF_003327025.1_ASM332702v1_genomic
GCF_003327055.1_ASM332705v1_genomic
GCF_003327065.1_ASM332706v1_genomic
GCF_003327105.1_ASM332710v1_genomic
GCF_003327115.1_ASM332711v1_genomic
GCF_003327145.1_ASM332714v1_genomic
GCF_003327165.1_ASM332716v1_genomic
GCF_003327185.1_ASM332718v1_genomic
GCF_003327215.1_ASM332721v1_genomic
GCF_003327265.1_ASM332726v1_genomic
GCF_003327325.1_ASM332732v1_genomic
GCF_003327345.1_ASM332734v1_genomic
GCF_003327365.1_ASM332736v1_genomic
GCF_003327385.1_ASM332738v1_genomic
GCF_003328455.1_ASM332845v1_genomic
GCF_003456975.1_ASM345697v1_genomic
GCF_003457015.1_ASM345701v1_genomic
GCF_003457035.1_ASM345703v1_genomic
GCF_003481905.1_ASM348190v1_genomic
GCF_003481965.1_ASM348196v1_genomic
GCF_003482035.1_ASM348203v1_genomic
GCF_003482065.1_ASM348206v1_genomic
GCF_003482125.1_ASM348212v1_genomic
GCF_003482165.1_ASM348216v1_genomic
GCF_003482225.1_ASM348222v1_genomic
GCF_003482255.1_ASM348225v1_genomic
GCF_003482305.1_ASM348230v1_genomic
GCF_003482325.1_ASM348232v1_genomic
GCF_003482345.1_ASM348234v1_genomic

GCF_003482365.1_ASM348236v1_genomic
GCF_003490105.1_ASM349010v1_genomic
GCF_003597775.1_ASM359777v1_genomic
GCF_003597795.1_ASM359779v1_genomic
GCF_003597815.1_ASM359781v1_genomic
GCF_003597835.1_ASM359783v1_genomic
GCF_003597855.1_ASM359785v1_genomic
GCF_003597875.1_ASM359787v1_genomic
GCF_003597895.1_ASM359789v1_genomic
GCF_003597915.1_ASM359791v1_genomic
GCF_003625425.1_ASM362542v1_genomic
GCF_003697205.1_ASM369720v1_genomic
GCF_003697225.1_ASM369722v1_genomic
GCF_003697245.1_ASM369724v1_genomic
GCF_003862565.1_ASM386256v1_genomic
GCF_003862575.1_ASM386257v1_genomic
GCF_003862595.1_ASM386259v1_genomic
GCF_003862635.1_ASM386263v1_genomic
GCF_003862655.1_ASM386265v1_genomic
GCF_003862665.1_ASM386266v1_genomic
GCF_003862685.1_ASM386268v1_genomic
GCF_003862705.1_ASM386270v1_genomic
GCF_003862755.1_ASM386275v1_genomic
GCF_003862785.1_ASM386278v1_genomic
GCF_003862815.1_ASM386281v1_genomic
GCF_003862835.1_ASM386283v1_genomic
GCF_003862855.1_ASM386285v1_genomic
GCF_003862885.1_ASM386288v1_genomic
GCF_003862935.1_ASM386293v1_genomic
GCF_003863005.1_ASM386300v1_genomic
GCF_003863035.1_ASM386303v1_genomic
GCF_003863045.1_ASM386304v1_genomic
GCF_003863055.1_ASM386305v1_genomic
GCF_003863075.1_ASM386307v1_genomic
GCF_003863105.1_ASM386310v1_genomic
GCF_003863135.1_ASM386313v1_genomic
GCF_003863145.1_ASM386314v1_genomic
GCF_003863235.1_ASM386323v1_genomic
GCF_003863265.1_ASM386326v1_genomic
GCF_003863275.1_ASM386327v1_genomic
GCF_003863305.1_ASM386330v1_genomic
GCF_003932635.1_ASM393263v1_genomic
GCF_003932645.1_ASM393264v1_genomic
GCF_003932675.1_ASM393267v1_genomic
GCF_003932695.1_ASM393269v1_genomic
GCF_004313745.1_ASM431374v1_genomic
GCF_004313985.1_ASM431398v1_genomic
GCF_004314245.1_ASM431424v1_genomic
GCF_004314265.1_ASM431426v1_genomic
GCF_004314365.1_ASM431436v1_genomic
GCF_004314385.1_ASM431438v1_genomic
GCF_004314405.1_ASM431440v1_genomic
GCF_004314425.1_ASM431442v1_genomic
GCF_004314445.1_ASM431444v1_genomic
GCF_004314485.1_ASM431448v1_genomic
GCF_004314505.1_ASM431450v1_genomic
GCF_004314525.1_ASM431452v1_genomic

GCF_004314645.1_ASM431464v1_genomic
GCF_004314725.1_ASM431472v1_genomic
GCF_004314825.1_ASM431482v1_genomic
GCF_004315105.1_ASM431510v1_genomic
GCF_004315245.1_ASM431524v1_genomic
GCF_004315485.1_ASM431548v1_genomic
GCF_004315505.1_ASM431550v1_genomic
GCF_004315705.1_ASM431570v1_genomic
GCF_004315785.1_ASM431578v1_genomic
GCF_004315865.1_ASM431586v1_genomic
GCF_004315945.1_ASM431594v1_genomic
GCF_004316025.1_ASM431602v1_genomic
GCF_004316085.1_ASM431608v1_genomic
GCF_004316125.1_ASM431612v1_genomic
GCF_004316145.1_ASM431614v1_genomic
GCF_004316165.1_ASM431616v1_genomic
GCF_004316245.1_ASM431624v1_genomic
GCF_004316385.1_ASM431638v1_genomic
GCF_004316405.1_ASM431640v1_genomic
GCF_004316425.1_ASM431642v1_genomic
GCF_004316445.1_ASM431644v1_genomic
GCF_004316465.1_ASM431646v1_genomic
GCF_004316485.1_ASM431648v1_genomic
GCF_004316785.1_ASM431678v1_genomic
GCF_004316805.1_ASM431680v1_genomic
GCF_004316825.1_ASM431682v1_genomic
GCF_004316845.1_ASM431684v1_genomic
GCF_004316885.1_ASM431688v1_genomic
GCF_004316905.1_ASM431690v1_genomic
GCF_004316925.1_ASM431692v1_genomic
GCF_004317025.1_ASM431702v1_genomic
GCF_004318205.1_ASM431820v1_genomic
GCF_004319425.1_ASM431942v1_genomic
GCF_004684575.1_ASM468457v1_genomic
GCF_004684585.1_ASM468458v1_genomic
GCF_004684595.1_ASM468459v1_genomic
GCF_004684655.1_ASM468465v1_genomic
GCF_004684675.1_ASM468467v1_genomic
GCF_004684745.1_ASM468474v1_genomic
GCF_004684755.1_ASM468475v1_genomic
GCF_005502205.1_ASM550220v1_genomic
GCF_006379335.1_ASM637933v1_genomic
GCF_006379355.1_ASM637935v1_genomic
GCF_006380825.1_ASM638082v1_genomic
GCF_006380835.1_ASM638083v1_genomic
GCF_006380875.1_ASM638087v1_genomic
GCF_006380895.1_ASM638089v1_genomic
GCF_006380905.1_ASM638090v1_genomic
GCF_006380915.1_ASM638091v1_genomic
GCF_006380955.1_ASM638095v1_genomic
GCF_006380975.1_ASM638097v1_genomic
GCF_006380995.1_ASM638099v1_genomic
GCF_006381005.1_ASM638100v1_genomic
GCF_006381025.1_ASM638102v1_genomic
GCF_006381045.1_ASM638104v1_genomic
GCF_006381075.1_ASM638107v1_genomic
GCF_006381095.1_ASM638109v1_genomic

GCF_006381115.1_ASM638111v1_genomic
GCF_006408235.1_ASM640823v1_genomic
GCF_006408255.1_ASM640825v1_genomic
GCF_006408285.1_ASM640828v1_genomic
GCF_006408325.1_ASM640832v1_genomic
GCF_006408335.1_ASM640833v1_genomic
GCF_006408375.1_ASM640837v1_genomic
GCF_006408415.1_ASM640841v1_genomic
GCF_006408435.1_ASM640843v1_genomic
GCF_006454545.1_ASM645454v1_genomic
GCF_006454555.1_ASM645455v1_genomic
GCF_006454575.1_ASM645457v1_genomic
GCF_007000295.1_ASM700029v1_genomic
GCF_007000385.1_ASM700038v1_genomic
GCF_007000395.1_ASM700039v1_genomic
GCF_007000425.1_ASM700042v1_genomic
GCF_007000435.1_ASM700043v1_genomic
GCF_007000525.1_ASM700052v1_genomic
GCF_007000575.1_ASM700057v1_genomic
GCF_007000645.1_ASM700064v1_genomic
GCF_007001125.1_ASM700112v1_genomic
GCF_007001285.1_ASM700128v1_genomic
GCF_007001365.1_ASM700136v1_genomic
GCF_007001555.1_ASM700155v1_genomic
GCF_007001585.1_ASM700158v1_genomic
GCF_007001605.1_ASM700160v1_genomic
GCF_007001645.1_ASM700164v1_genomic
GCF_007001665.1_ASM700166v1_genomic
GCF_007001725.1_ASM700172v1_genomic
GCF_007001815.1_ASM700181v1_genomic
GCF_007001865.1_ASM700186v1_genomic
GCF_007001905.1_ASM700190v1_genomic
GCF_007001945.1_ASM700194v1_genomic
GCF_007001975.1_ASM700197v1_genomic
GCF_007002035.1_ASM700203v1_genomic
GCF_007002055.1_ASM700205v1_genomic
GCF_007002095.1_ASM700209v1_genomic
GCF_007002145.1_ASM700214v1_genomic
GCF_007002155.1_ASM700215v1_genomic
GCF_007002165.1_ASM700216v1_genomic
GCF_007002205.1_ASM700220v1_genomic
GCF_007002215.1_ASM700221v1_genomic
GCF_007002235.1_ASM700223v1_genomic
GCF_007002255.1_ASM700225v1_genomic
GCF_007002265.1_ASM700226v1_genomic
GCF_007002285.1_ASM700228v1_genomic
GCF_007002325.1_ASM700232v1_genomic
GCF_007002345.1_ASM700234v1_genomic
GCF_007002365.1_ASM700236v1_genomic
GCF_007002385.1_ASM700238v1_genomic
GCF_007002455.1_ASM700245v1_genomic
GCF_007002635.1_ASM700263v1_genomic
GCF_007002665.1_ASM700266v1_genomic
GCF_007113935.1_ASM711393v1_genomic
GCF_007113975.1_ASM711397v1_genomic
GCF_007114005.1_ASM711400v1_genomic
GCF_007114015.1_ASM711401v1_genomic

GCF_007114045.1_ASM711404v1_genomic
GCF_007114055.1_ASM711405v1_genomic
GCF_007114075.1_ASM711407v1_genomic
GCF_007114095.1_ASM711409v1_genomic
GCF_007114115.1_ASM711411v1_genomic
GCF_007114145.1_ASM711414v1_genomic
GCF_007114155.1_ASM711415v1_genomic
GCF_007114185.1_ASM711418v1_genomic
GCF_007114195.1_ASM711419v1_genomic
GCF_007114205.1_ASM711420v1_genomic
GCF_007114225.1_ASM711422v1_genomic
GCF_007114235.1_ASM711423v1_genomic
GCF_007114285.1_ASM711428v1_genomic
GCF_007114305.1_ASM711430v1_genomic
GCF_007114315.1_ASM711431v1_genomic
GCF_007114325.1_ASM711432v1_genomic
GCF_007114335.1_ASM711433v1_genomic
GCF_007114385.1_ASM711438v1_genomic
GCF_007114395.1_ASM711439v1_genomic
GCF_007114405.1_ASM711440v1_genomic
GCF_007114415.1_ASM711441v1_genomic
GCF_007114465.1_ASM711446v1_genomic
GCF_007114485.1_ASM711448v1_genomic
GCF_007114495.1_ASM711449v1_genomic
GCF_007114515.1_ASM711451v1_genomic
GCF_007114535.1_ASM711453v1_genomic
GCF_007114555.1_ASM711455v1_genomic
GCF_007114585.1_ASM711458v1_genomic
GCF_007114595.1_ASM711459v1_genomic
GCF_007114605.1_ASM711460v1_genomic
GCF_007114645.1_ASM711464v1_genomic
GCF_007114655.1_ASM711465v1_genomic
GCF_007114685.1_ASM711468v1_genomic
GCF_007114695.1_ASM711469v1_genomic
GCF_007114705.1_ASM711470v1_genomic
GCF_007114725.1_ASM711472v1_genomic
GCF_007114735.1_ASM711473v1_genomic
GCF_007114785.1_ASM711478v1_genomic
GCF_007114805.1_ASM711480v1_genomic
GCF_007114815.1_ASM711481v1_genomic
GCF_007114825.1_ASM711482v1_genomic
GCF_007114835.1_ASM711483v1_genomic
GCF_007114885.1_ASM711488v1_genomic
GCF_007114905.1_ASM711490v1_genomic
GCF_007114915.1_ASM711491v1_genomic
GCF_007114935.1_ASM711493v1_genomic
GCF_007114945.1_ASM711494v1_genomic
GCF_007114985.1_ASM711498v1_genomic
GCF_007114995.1_ASM711499v1_genomic
GCF_008245165.1_ASM824516v1_genomic
GCF_008579085.1_ASM857908v1_genomic
GCF_009362915.1_ASM936291v1_genomic
GCF_009730495.1_ASM973049v1_genomic
GCF_009867095.1_ASM986709v1_genomic
GCF_009903385.1_ASM990338v1_genomic
GCF_009903395.1_ASM990339v1_genomic
GCF_009903465.1_ASM990346v1_genomic

GCF_009903485.1_ASM990348v1_genomic
GCF_009903525.1_ASM990352v1_genomic
GCF_009903535.1_ASM990353v1_genomic
GCF_009903545.1_ASM990354v1_genomic
GCF_011045515.1_ASM1104551v1_genomic
GCF_011602815.1_ASM1160281v1_genomic
GCF_012641505.1_ASM1264150v1_genomic
GCF_012952655.1_ASM1295265v1_genomic
GCF_014236775.1_ASM1423677v1_genomic
GCF_014333625.1_ASM1433362v1_genomic
GCF_015238635.1_ASM1523863v1_genomic
GCF_015351775.1_ASM1535177v1_genomic
GCF_015351815.1_ASM1535181v1_genomic
GCF_015351875.1_ASM1535187v1_genomic
GCF_015351965.1_ASM1535196v1_genomic
GCF_015352015.1_ASM1535201v1_genomic
GCF_015352035.1_ASM1535203v1_genomic
GCF_015352045.1_ASM1535204v1_genomic
GCF_015352065.1_ASM1535206v1_genomic
GCF_015352115.1_ASM1535211v1_genomic
GCF_015352165.1_ASM1535216v1_genomic
GCF_015352175.1_ASM1535217v1_genomic
GCF_015352235.1_ASM1535223v1_genomic
GCF_015352285.1_ASM1535228v1_genomic
GCF_015352395.1_ASM1535239v1_genomic
GCF_015686615.1_ASM1568661v1_genomic
GCF_015686625.1_ASM1568662v1_genomic
GCF_015732535.1_ASM1573253v1_genomic
GCF_015732555.1_ASM1573255v1_genomic
GCF_016093595.1_ASM1609359v1_genomic
GCF_016093775.1_ASM1609377v1_genomic
GCF_016093825.1_ASM1609382v1_genomic
GCF_016093925.1_ASM1609392v1_genomic
GCF_016093965.1_ASM1609396v1_genomic
GCF_016093995.1_ASM1609399v1_genomic
GCF_016094015.1_ASM1609401v1_genomic
GCF_016094025.1_ASM1609402v1_genomic
GCF_016094055.1_ASM1609405v1_genomic
GCF_016094095.1_ASM1609409v1_genomic
GCF_016094135.1_ASM1609413v1_genomic
GCF_016094145.1_ASM1609414v1_genomic
GCF_016094175.1_ASM1609417v1_genomic
GCF_016094195.1_ASM1609419v1_genomic
GCF_016094205.1_ASM1609420v1_genomic
GCF_016094215.1_ASM1609421v1_genomic
GCF_016094245.1_ASM1609424v1_genomic
GCF_016094275.1_ASM1609427v1_genomic
GCF_016094285.1_ASM1609428v1_genomic
GCF_016094335.1_ASM1609433v1_genomic
GCF_016766955.1_ASM1676695v1_genomic
GCF_016766975.1_ASM1676697v1_genomic
GCF_016766995.1_ASM1676699v1_genomic
GCF_016767015.1_ASM1676701v1_genomic
GCF_016767035.1_ASM1676703v1_genomic
GCF_016767055.1_ASM1676705v1_genomic
GCF_016767095.1_ASM1676709v1_genomic
GCF_016767115.1_ASM1676711v1_genomic

GCF_016767135.1_ASM1676713v1_genomic
GCF_016985475.1_ASM1698547v1_genomic
GCF_017310655.1_ASM1731065v1_genomic
GCF_017310685.1_ASM1731068v1_genomic
GCF_017310705.1_ASM1731070v1_genomic
GCF_017310715.1_ASM1731071v1_genomic
GCF_017310745.1_ASM1731074v1_genomic
GCF_017310775.1_ASM1731077v1_genomic
GCF_017310805.1_ASM1731080v1_genomic
GCF_017310825.1_ASM1731082v1_genomic
GCF_017310845.1_ASM1731084v1_genomic
GCF_017310865.1_ASM1731086v1_genomic
GCF_017310885.1_ASM1731088v1_genomic
GCF_017310895.1_ASM1731089v1_genomic
GCF_017310915.1_ASM1731091v1_genomic
GCF_017310935.1_ASM1731093v1_genomic
GCF_017310985.1_ASM1731098v1_genomic
GCF_017310995.1_ASM1731099v1_genomic
GCF_017311045.1_ASM1731104v1_genomic
GCF_017311065.1_ASM1731106v1_genomic
GCF_017311085.1_ASM1731108v1_genomic
GCF_018255775.1_ASM1825577v1_genomic
GCF_018263895.1_ASM1826389v1_genomic
GCF_018263955.1_ASM1826395v1_genomic
GCF_018263995.1_ASM1826399v1_genomic
GCF_018264005.1_ASM1826400v1_genomic
GCF_018264015.1_ASM1826401v1_genomic
GCF_018264045.1_ASM1826404v1_genomic
GCF_018413615.1_ASM1841361v1_genomic
GCF_018603395.1_ASM1860339v1_genomic
GCF_018603455.1_ASM1860345v1_genomic
GCF_018603475.1_ASM1860347v1_genomic
GCF_018884605.1_ASM1888460v1_genomic
GCF_018884625.1_ASM1888462v1_genomic
GCF_018884645.1_ASM1888464v1_genomic
GCF_018884665.1_ASM1888466v1_genomic
GCF_018884685.1_ASM1888468v1_genomic
GCF_018884705.1_ASM1888470v1_genomic
GCF_018884725.1_ASM1888472v1_genomic
GCF_018884745.1_ASM1888474v1_genomic
GCF_018884765.1_ASM1888476v1_genomic
GCF_018884785.1_ASM1888478v1_genomic
GCF_018884805.1_ASM1888480v1_genomic
GCF_018884825.1_ASM1888482v1_genomic
GCF_018884845.1_ASM1888484v1_genomic
GCF_018884865.1_ASM1888486v1_genomic
GCF_018884885.1_ASM1888488v1_genomic
GCF_018884905.1_ASM1888490v1_genomic
GCF_018884925.1_ASM1888492v1_genomic
GCF_018884945.1_ASM1888494v1_genomic
GCF_018884965.1_ASM1888496v1_genomic
GCF_018884985.1_ASM1888498v1_genomic
GCF_018885005.1_ASM1888500v1_genomic
GCF_018885025.1_ASM1888502v1_genomic
GCF_018885045.1_ASM1888504v1_genomic
GCF_018885065.1_ASM1888506v1_genomic
GCF_018885085.1_ASM1888508v1_genomic

GCF_018885105.1_ASM1888510v1_genomic
GCF_018917125.1_ASM1891712v1_genomic
GCF_019067085.1_ASM1906708v1_genomic
GCF_019095765.1_ASM1909576v1_genomic
GCF_019880625.1_ASM1988062v1_genomic
GCF_019931025.1_ASM1993102v1_genomic
GCF_020097215.1_ASM2009721v1_genomic
GCF_020295125.1_ASM2029512v1_genomic
GCF_020295155.1_ASM2029515v1_genomic
GCF_020295185.1_ASM2029518v1_genomic
GCF_020295225.1_ASM2029522v1_genomic
GCF_020295245.1_ASM2029524v1_genomic
GCF_020295265.1_ASM2029526v1_genomic
GCF_020295305.1_ASM2029530v1_genomic
GCF_020297505.1_ASM2029750v1_genomic
GCF_020297525.1_ASM2029752v1_genomic
GCF_020341515.1_ASM2034151v1_genomic
GCF_021378415.1_ASM2137841v1_genomic
GCF_021532205.1_ASM2153220v1_genomic
GCF_022458765.1_ASM2245876v1_genomic
GCF_022458775.1_ASM2245877v1_genomic
GCF_022458805.1_ASM2245880v1_genomic
GCF_022458815.1_ASM2245881v1_genomic
GCF_022458845.1_ASM2245884v1_genomic
GCF_022695655.1_ASM2269565v1_genomic
GCF_022845595.1_ASM2284559v1_genomic
GCF_022970035.1_ASM2297003v1_genomic
GCF_022970075.1_ASM2297007v1_genomic
GCF_022970095.1_ASM2297009v1_genomic
GCF_022970115.1_ASM2297011v1_genomic
GCF_022970135.1_ASM2297013v1_genomic
GCF_022970155.1_ASM2297015v1_genomic
GCF_022970175.1_ASM2297017v1_genomic
GCF_022970195.1_ASM2297019v1_genomic
GCF_022970215.1_ASM2297021v1_genomic
GCF_022970235.1_ASM2297023v1_genomic
GCF_022970255.1_ASM2297025v1_genomic
GCF_022970275.1_ASM2297027v1_genomic
GCF_022970295.1_ASM2297029v1_genomic
GCF_022970315.1_ASM2297031v1_genomic
GCF_022970335.1_ASM2297033v1_genomic
GCF_022970355.1_ASM2297035v1_genomic
GCF_022970495.1_ASM2297049v1_genomic
GCF_024260025.1_ASM2426002v1_genomic
GCF_024260125.1_ASM2426012v1_genomic
GCF_024260185.1_ASM2426018v1_genomic
GCF_024260205.1_ASM2426020v1_genomic
GCF_024260215.1_ASM2426021v1_genomic
GCF_024260245.1_ASM2426024v1_genomic
GCF_024260265.1_ASM2426026v1_genomic
GCF_024260285.1_ASM2426028v1_genomic
GCF_024260295.1_ASM2426029v1_genomic
GCF_024260325.1_ASM2426032v1_genomic
GCF_024260345.1_ASM2426034v1_genomic
GCF_024260365.1_ASM2426036v1_genomic
GCF_024260385.1_ASM2426038v1_genomic
GCF_024260405.1_ASM2426040v1_genomic

GCF_024260425.1_ASM2426042v1_genomic
GCF_024260445.1_ASM2426044v1_genomic
GCF_024260465.1_ASM2426046v1_genomic
GCF_024260485.1_ASM2426048v1_genomic
GCF_024260515.1_ASM2426051v1_genomic
GCF_024262265.1_ASM2426226v1_genomic
GCF_024262305.1_ASM2426230v1_genomic
GCF_024262325.1_ASM2426232v1_genomic
GCF_024262385.1_ASM2426238v1_genomic
GCF_024262405.1_ASM2426240v1_genomic
GCF_024399435.1_ASM2439943v1_genomic
GCF_024451205.1_ASM2445120v1_genomic
GCF_900005755.1_CD05HE1_ppCDHM1_genomic
GCF_900095895.1_ASM90009589v1_genomic
GCF_900095905.1_ASM90009590v1_genomic
GCF_900095915.1_ASM90009591v1_genomic
GCF_900095925.1_ASM90009592v1_genomic
GCF_900095935.1_ASM90009593v1_genomic
GCF_900095945.1_ASM90009594v1_genomic
GCF_900095955.1_ASM90009595v1_genomic
GCF_900095965.1_ASM90009596v1_genomic
GCF_900095975.1_ASM90009597v1_genomic
GCF_900095985.1_ASM90009598v1_genomic
GCF_900095995.1_ASM90009599v1_genomic
GCF_900096005.1_ASM90009600v1_genomic
GCF_900096015.1_ASM90009601v1_genomic
GCF_900096025.1_ASM90009602v1_genomic
GCF_900096035.1_ASM90009603v1_genomic
GCF_900096045.1_ASM90009604v1_genomic
GCF_900096055.1_ASM90009605v1_genomic
GCF_900096065.1_ASM90009606v1_genomic
GCF_900096075.1_ASM90009607v1_genomic
GCF_900096085.1_ASM90009608v1_genomic
GCF_900096095.1_ASM90009609v1_genomic
GCF_900096105.1_ASM90009610v1_genomic
GCF_900096115.1_ASM90009611v1_genomic
GCF_900096125.1_ASM90009612v1_genomic
GCF_900096135.1_ASM90009613v1_genomic
GCF_900096145.1_ASM90009614v1_genomic
GCF_900096155.1_ASM90009615v1_genomic
GCF_900096165.1_ASM90009616v1_genomic
GCF_900096175.1_ASM90009617v1_genomic
GCF_900096185.1_ASM90009618v1_genomic
GCF_900096195.1_ASM90009619v1_genomic
GCF_900096205.1_ASM90009620v1_genomic
GCF_900096215.1_ASM90009621v1_genomic
GCF_900096225.1_ASM90009622v1_genomic
GCF_900096235.1_ASM90009623v1_genomic
GCF_900096245.1_ASM90009624v1_genomic
GCF_900096255.1_ASM90009625v1_genomic
GCF_900096265.1_ASM90009626v1_genomic
GCF_900096275.1_ASM90009627v1_genomic
GCF_900096285.1_ASM90009628v1_genomic
GCF_900096295.1_ASM90009629v1_genomic
GCF_900096305.1_ASM90009630v1_genomic
GCF_900096315.1_ASM90009631v1_genomic
GCF_900096325.1_ASM90009632v1_genomic

GCF_900096335.1_ASM90009633v1_genomic
GCF_900096345.1_ASM90009634v1_genomic
GCF_900096355.1_ASM90009635v1_genomic
GCF_900096365.1_ASM90009636v1_genomic
GCF_900096375.1_ASM90009637v1_genomic
GCF_900096385.1_ASM90009638v1_genomic
GCF_900096395.1_ASM90009639v1_genomic
GCF_900096405.1_ASM90009640v1_genomic
GCF_900096415.1_ASM90009641v1_genomic
GCF_900096425.1_ASM90009642v1_genomic
GCF_900096435.1_ASM90009643v1_genomic
GCF_900096445.1_ASM90009644v1_genomic
GCF_900096455.1_ASM90009645v1_genomic
GCF_900096465.1_ASM90009646v1_genomic
GCF_900096475.1_ASM90009647v1_genomic
GCF_900164015.1_16780_8_22_genomic
GCF_900164025.1_16780_8_13_genomic
GCF_900164045.1_16780_8_27_genomic
GCF_900164055.1_16780_8_26_genomic
GCF_900164065.1_16780_8_20_genomic
GCF_900164075.1_16780_8_14_genomic
GCF_900164085.1_16780_8_31_genomic
GCF_900164095.1_16780_8_53_genomic
GCF_900164105.1_16780_8_28_genomic
GCF_900164115.1_16780_8_32_genomic
GCF_900164125.1_16780_8_49_genomic
GCF_900164135.1_16780_8_47_genomic
GCF_900164145.1_16780_8_19_genomic
GCF_900164155.1_16780_8_7_genomic
GCF_900164165.1_16780_8_51_genomic
GCF_900164175.1_16780_8_21_genomic
GCF_900164185.1_16780_8_48_genomic
GCF_900164195.1_16780_8_63_genomic
GCF_900164205.1_16780_8_8_genomic
GCF_900164215.1_16780_8_30_genomic
GCF_900164225.1_16780_8_15_genomic
GCF_900164245.1_16780_8_50_genomic
GCF_900164255.1_16780_8_16_genomic
GCF_900164265.1_16780_8_52_genomic
GCF_900164275.1_16780_8_5_genomic
GCF_900164285.1_16780_8_17_genomic
GCF_900164295.1_16780_8_23_genomic
GCF_900164305.1_16780_8_6_genomic
GCF_900164325.1_16780_8_9_genomic
GCF_900164335.1_16780_8_55_genomic
GCF_900164345.1_16780_8_61_genomic
GCF_900164355.1_16780_8_29_genomic
GCF_900164375.1_16780_8_12_genomic
GCF_900164395.1_16780_8_25_genomic
GCF_900164405.1_16780_8_56_genomic
GCF_900164415.1_16780_8_64_genomic
GCF_900164425.1_16780_8_62_genomic
GCF_900164435.1_16780_8_67_genomic
GCF_900164445.1_16780_8_58_genomic
GCF_900164455.1_16780_8_57_genomic
GCF_900164465.1_16780_8_60_genomic
GCF_900164475.1_16780_8_24_genomic

GCF_900164485.1_16780_8_59_genomic
GCF_900164495.1_16780_8_54_genomic
GCF_900164505.1_16780_8_68_genomic
GCF_900164515.1_16780_8_69_genomic
GCF_900164525.1_16780_8_76_genomic
GCF_900164535.1_16780_8_79_genomic
GCF_900164545.1_16780_8_80_genomic
GCF_900164555.1_16852_2_1_genomic
GCF_900164565.1_16780_8_94_genomic
GCF_900164575.1_16852_2_2_genomic
GCF_900164585.1_16852_2_3_genomic
GCF_900164595.1_16852_2_4_genomic
GCF_900164605.1_16852_2_5_genomic
GCF_900164615.1_16852_2_6_genomic
GCF_900164625.1_16852_2_10_genomic
GCF_900164635.1_16852_2_13_genomic
GCF_900164645.1_16852_2_11_genomic
GCF_900164655.1_16852_2_14_genomic
GCF_900164665.1_16852_2_16_genomic
GCF_900164675.1_16852_2_17_genomic
GCF_900164685.1_16852_2_19_genomic
GCF_900164695.1_16852_2_23_genomic
GCF_900164705.1_16852_2_27_genomic
GCF_900164715.1_16852_2_35_genomic
GCF_900164725.1_16852_2_36_genomic
GCF_900164735.1_16852_2_37_genomic
GCF_900164745.1_16852_2_38_genomic
GCF_900164755.1_16852_2_39_genomic
GCF_900164765.1_16852_2_41_genomic
GCF_900164775.1_16852_2_42_genomic
GCF_900164785.1_16852_2_43_genomic
GCF_900164795.1_16852_2_45_genomic
GCF_900164805.1_16852_2_46_genomic
GCF_900164815.1_16852_2_47_genomic
GCF_900164825.1_16852_2_50_genomic
GCF_900164835.1_16852_2_49_genomic
GCF_900164845.1_16852_2_48_genomic
GCF_900164855.1_16852_2_66_genomic
GCF_900164865.1_16852_2_65_genomic
GCF_900164875.1_16852_2_67_genomic
GCF_900164885.1_16852_2_71_genomic
GCF_900164895.1_16852_2_69_genomic
GCF_900164905.1_16852_2_72_genomic
GCF_900164915.1_16852_2_73_genomic
GCF_900164925.1_16852_2_75_genomic
GCF_900164935.1_16852_2_74_genomic
GCF_900164945.1_16852_2_83_genomic
GCF_900164955.1_16852_2_82_genomic
GCF_900164965.1_16852_2_84_genomic
GCF_900164975.1_16852_2_85_genomic
GCF_900164985.1_16852_2_86_genomic
GCF_900164995.1_16852_2_87_genomic
GCF_900165005.1_16852_2_88_genomic
GCF_900165015.1_16852_2_89_genomic
GCF_900165025.1_16780_8_1_genomic
GCF_900165035.1_16780_8_2_genomic
GCF_900165045.1_16780_8_3_genomic

GCF_900165055.1_16780_8_4_genomic
GCF_900165065.1_16780_8_10_genomic
GCF_900165075.1_16780_8_11_genomic
GCF_900165085.1_16780_8_33_genomic
GCF_900165095.1_16780_8_34_genomic
GCF_900165105.1_16780_8_35_genomic
GCF_900165115.1_16780_8_36_genomic
GCF_900165125.1_16780_8_37_genomic
GCF_900165135.1_16780_8_38_genomic
GCF_900165145.1_16780_8_39_genomic
GCF_900165155.1_16780_8_40_genomic
GCF_900165165.1_16780_8_41_genomic
GCF_900165175.1_16780_8_42_genomic
GCF_900165185.1_16780_8_43_genomic
GCF_900165195.1_16780_8_44_genomic
GCF_900165205.1_16780_8_45_genomic
GCF_900165215.1_16780_8_46_genomic
GCF_900165225.1_16780_8_65_genomic
GCF_900165235.1_16780_8_66_genomic
GCF_900165245.1_16780_8_70_genomic
GCF_900165255.1_16780_8_71_genomic
GCF_900165265.1_16780_8_72_genomic
GCF_900165275.1_16780_8_73_genomic
GCF_900165285.1_16780_8_74_genomic
GCF_900165295.1_16780_8_78_genomic
GCF_900165305.1_16780_8_77_genomic
GCF_900165315.1_16780_8_75_genomic
GCF_900165325.1_16780_8_81_genomic
GCF_900165335.1_16780_8_82_genomic
GCF_900165345.1_16780_8_83_genomic
GCF_900165355.1_16780_8_84_genomic
GCF_900165365.1_16780_8_85_genomic
GCF_900165375.1_16780_8_86_genomic
GCF_900165385.1_16780_8_91_genomic
GCF_900165395.1_16780_8_90_genomic
GCF_900165405.1_16780_8_92_genomic
GCF_900165415.1_16780_8_89_genomic
GCF_900165425.1_16780_8_88_genomic
GCF_900165435.1_16780_8_93_genomic
GCF_900165455.1_16852_2_7_genomic
GCF_900165465.1_16852_2_8_genomic
GCF_900165475.1_16852_2_9_genomic
GCF_900165485.1_16852_2_12_genomic
GCF_900165495.1_16852_2_15_genomic
GCF_900165505.1_16852_2_20_genomic
GCF_900165515.1_16852_2_18_genomic
GCF_900165525.1_16852_2_21_genomic
GCF_900165535.1_16852_2_22_genomic
GCF_900165545.1_16852_2_24_genomic
GCF_900165555.1_16852_2_25_genomic
GCF_900165565.1_16852_2_26_genomic
GCF_900165575.1_16852_2_28_genomic
GCF_900165585.1_16852_2_29_genomic
GCF_900165595.1_16852_2_30_genomic
GCF_900165605.1_16852_2_31_genomic
GCF_900165615.1_16852_2_33_genomic
GCF_900165625.1_16852_2_32_genomic

GCF_900165635.1_16852_2_34_genomic
GCF_900165645.1_16852_2_51_genomic
GCF_900165655.1_16852_2_52_genomic
GCF_900165665.1_16852_2_53_genomic
GCF_900165675.1_16852_2_54_genomic
GCF_900165685.1_16852_2_55_genomic
GCF_900165695.1_16852_2_56_genomic
GCF_900165705.1_16852_2_57_genomic
GCF_900165715.1_16852_2_58_genomic
GCF_900165725.1_16852_2_59_genomic
GCF_900165735.1_16852_2_60_genomic
GCF_900165745.1_16852_2_61_genomic
GCF_900165755.1_16852_2_62_genomic
GCF_900165765.1_16852_2_63_genomic
GCF_900165775.1_16852_2_64_genomic
GCF_900165785.1_16852_2_76_genomic
GCF_900165795.1_16852_2_77_genomic
GCF_900165805.1_16852_2_78_genomic
GCF_900165815.1_16852_2_79_genomic
GCF_900165825.1_16852_2_80_genomic
GCF_900165835.1_16852_2_81_genomic
GCF_900165845.1_16852_2_90_genomic
GCF_900165855.1_16852_2_91_genomic
GCF_900165865.1_16852_2_92_genomic
GCF_900165875.1_16852_2_93_genomic
GCF_900165885.1_11670_1_66_genomic
GCF_900165895.1_11670_1_27_genomic
GCF_900165905.1_11670_1_18_genomic
GCF_900165915.1_11670_1_56_genomic
GCF_900165925.1_11670_1_28_genomic
GCF_900165935.1_11670_1_29_genomic
GCF_900165945.1_11670_1_68_genomic
GCF_900165955.1_11670_1_2_genomic
GCF_900165965.1_11670_1_72_genomic
GCF_900165975.1_11670_1_4_genomic
GCF_900165985.1_11670_1_65_genomic
GCF_900165995.1_11670_1_1_genomic
GCF_900166005.1_11670_1_63_genomic
GCF_900166015.1_11670_1_32_genomic
GCF_900166025.1_11670_1_52_genomic
GCF_900166035.1_11670_1_57_genomic
GCF_900166045.1_11670_1_6_genomic
GCF_900166055.1_11670_1_30_genomic
GCF_900166065.1_11670_1_70_genomic
GCF_900166075.1_11670_1_69_genomic
GCF_900166085.1_11670_1_25_genomic
GCF_900166095.1_11670_1_71_genomic
GCF_900166105.1_11670_1_42_genomic
GCF_900166115.1_11670_1_48_genomic
GCF_900166125.1_11670_1_5_genomic
GCF_900166135.1_11670_1_26_genomic
GCF_900166145.1_11670_1_34_genomic
GCF_900166155.1_11670_1_22_genomic
GCF_900166165.1_11670_1_40_genomic
GCF_900166175.1_11670_1_45_genomic
GCF_900166185.1_11670_1_54_genomic
GCF_900166195.1_11670_1_36_genomic

GCF_900166205.1_11670_1_24_genomic
GCF_900166215.1_11670_1_55_genomic
GCF_900166225.1_11670_1_53_genomic
GCF_900166235.1_11670_1_7_genomic
GCF_900166245.1_11670_1_23_genomic
GCF_900166255.1_11670_1_11_genomic
GCF_900166265.1_11670_1_67_genomic
GCF_900166275.1_11670_1_58_genomic
GCF_900166285.1_11670_1_61_genomic
GCF_900166295.1_11670_1_46_genomic
GCF_900166305.1_11670_1_10_genomic
GCF_900166315.1_11670_1_21_genomic
GCF_900166325.1_11670_1_47_genomic
GCF_900166335.1_11670_1_41_genomic
GCF_900166345.1_11670_1_38_genomic
GCF_900166355.1_11670_1_15_genomic
GCF_900166365.1_11670_1_19_genomic
GCF_900166375.1_11670_1_50_genomic
GCF_900166385.1_11670_1_33_genomic
GCF_900166395.1_11670_1_37_genomic
GCF_900166405.1_11670_1_17_genomic
GCF_900166415.1_11670_1_51_genomic
GCF_900166425.1_11670_1_62_genomic
GCF_900166435.1_11670_1_39_genomic
GCF_900166445.1_11670_1_12_genomic
GCF_900166455.1_11670_1_60_genomic
GCF_900166465.1_11670_1_14_genomic
GCF_900166475.1_11670_1_13_genomic
GCF_900166485.1_11670_1_49_genomic
GCF_900166495.1_11670_1_44_genomic
GCF_900166505.1_11670_1_9_genomic
GCF_900166515.1_11670_1_59_genomic
GCF_900166525.1_11670_1_73_genomic
GCF_900166535.1_11670_1_16_genomic
GCF_900166545.1_11670_1_35_genomic
GCF_900166555.1_11670_1_3_genomic
GCF_900166565.1_11670_1_64_genomic
GCF_900167585.1_16852_2_94_genomic
GCF_900167595.1_16780_8_87_genomic
GCF_900167615.1_11670_1_20_genomic
GCF_900167625.1_16852_2_68_genomic
GCF_900243115.1_57267_genomic
GCF_900243155.1_2402_genomic
GCF_900243175.1_J9965_genomic
GCF_900243325.1_R_11402_genomic
GCF_900243345.1_R_9385_genomic
GCF_900446965.1_45232_F01_genomic
GCF_900447005.1_45296_B02_genomic
GCF_900447055.1_45296_C02_genomic
GCF_900683845.1_4880_3_10_genomic
GCF_900683865.1_4880_3_3_genomic
GCF_900683905.1_4880_3_9_genomic
GCF_900683915.1_4880_5_1_genomic
GCF_900683925.1_4880_5_10_genomic
GCF_900683935.1_4880_5_11_genomic
GCF_900683945.1_4880_5_2_genomic
GCF_900684125.1_4995_1_11_genomic

GCF_900684135.1_4995_1_2_genomic
GCF_900684165.1_4995_1_6_genomic
GCF_900684305.1_4995_2_8_genomic
GCF_900685245.1_5352_5_1_genomic
GCF_900685255.1_5352_3_9_genomic
GCF_900685285.1_5352_5_3_genomic
GCF_900685585.1_5550_3_11_genomic
GCF_900685905.1_5550_7_5_genomic
GCF_900686325.1_5578_5_11_genomic
GCF_900686855.1_5673_6_4_genomic
GCF_900687005.1_5673_7_5_genomic
GCF_900687115.1_5789_3_4_genomic
GCF_900687125.1_5789_3_2_genomic
GCF_900687235.1_6437_3_19_genomic
GCF_900687265.1_6437_3_16_genomic
GCF_900687285.1_6437_3_15_genomic
GCF_900687405.1_6437_4_10_genomic
GCF_900687475.1_6437_4_4_genomic
GCF_900687565.1_7614_4_51_genomic
GCF_900687575.1_7614_4_57_genomic
GCF_900687595.1_7614_4_55_genomic
GCF_900687605.1_7614_4_59_genomic
GCF_900687625.1_7614_4_62_genomic
GCF_900687645.1_7614_4_67_genomic
GCF_900687655.1_7614_4_63_genomic
GCF_900687665.1_7614_4_66_genomic
GCF_900687715.1_7614_4_74_genomic
GCF_900687725.1_7614_4_70_genomic
GCF_900687775.1_7614_4_49_genomic
GCF_900687785.1_7614_4_76_genomic
GCF_900687825.1_7614_4_75_genomic
GCF_900687915.1_7614_7_12_genomic
GCF_900687925.1_7614_7_1_genomic
GCF_900687955.1_7614_4_69_genomic
GCF_900687965.1_7614_7_16_genomic
GCF_900687975.1_7614_7_15_genomic
GCF_900688075.1_7614_7_2_genomic
GCF_900688085.1_7614_7_25_genomic
GCF_900688105.1_7614_7_30_genomic
GCF_900688195.1_7614_7_31_genomic
GCF_900688205.1_7614_7_32_genomic
GCF_900688245.1_7614_7_44_genomic
GCF_900688305.1_7614_7_39_genomic
GCF_900688315.1_7614_7_38_genomic
GCF_900688445.1_8080_1_15_genomic
GCF_900688505.1_8080_1_23_genomic
GCF_900688525.1_8080_1_21_genomic
GCF_900688535.1_8080_1_25_genomic
GCF_900688565.1_7614_7_47_genomic
GCF_900688665.1_7614_7_48_genomic
GCF_900688705.1_8080_1_16_genomic
GCF_900688715.1_8080_1_38_genomic
GCF_900688725.1_8080_1_18_genomic
GCF_900688735.1_8080_1_41_genomic
GCF_900688755.1_8080_1_17_genomic
GCF_900688775.1_8080_1_44_genomic
GCF_900688815.1_8080_1_47_genomic

GCF_900688835.1_8080_1_46_genomic
GCF_900688855.1_8080_1_37_genomic
GCF_900688875.1_8080_1_54_genomic
GCF_900688885.1_8080_1_6_genomic
GCF_900688895.1_8080_1_51_genomic
GCF_900688915.1_8080_1_9_genomic
GCF_900688925.1_8080_2_10_genomic
GCF_900688965.1_8080_2_17_genomic
GCF_900688975.1_8080_2_12_genomic
GCF_900689015.1_8080_2_11_genomic
GCF_900689035.1_8080_2_14_genomic
GCF_900689055.1_8080_2_20_genomic
GCF_900689405.1_8080_2_57_genomic
GCF_900689435.1_8080_2_2_genomic
GCF_900689445.1_8080_2_59_genomic
GCF_900689605.1_8080_2_74_genomic
GCF_900689705.1_8080_2_81_genomic
GCF_900689735.1_8080_2_84_genomic
GCF_900689745.1_8080_2_83_genomic
GCF_900689755.1_8080_2_89_genomic
GCF_900689765.1_8080_2_87_genomic
GCF_900689775.1_8080_2_9_genomic
GCF_900689825.1_8080_2_96_genomic
GCF_900689835.1_8080_2_94_genomic
GCF_900689855.1_8080_2_92_genomic
GCF_900689945.1_8140_6_18_genomic
GCF_900691155.1_9119_3_45_genomic
GCF_900691505.1_9119_3_80_genomic
GCF_900691745.1_9221_1_22_genomic
GCF_900691755.1_9221_1_25_genomic
GCF_900691805.1_9221_1_32_genomic
GCF_900691815.1_9221_1_34_genomic
GCF_900691835.1_9221_1_33_genomic
GCF_900691855.1_9221_1_41_genomic
GCF_900691875.1_9221_1_37_genomic
GCF_900691885.1_9221_1_42_genomic
GCF_900691915.1_9221_1_44_genomic
GCF_900691945.1_9221_1_46_genomic
GCF_900691955.1_9221_1_47_genomic
GCF_900691975.1_9221_1_35_genomic
GCF_900691985.1_9221_1_90_genomic
GCF_900692015.1_9221_1_49_genomic
GCF_900692035.1_9221_1_93_genomic
GCF_900692075.1_9221_6_17_genomic
GCF_900692085.1_9221_6_15_genomic
GCF_900692125.1_9221_6_13_genomic
GCF_900692135.1_9221_6_23_genomic
GCF_900692155.1_9221_6_20_genomic
GCF_900692175.1_9221_6_21_genomic
GCF_900692195.1_9221_6_30_genomic
GCF_900692205.1_9221_6_27_genomic
GCF_900692215.1_9221_6_29_genomic
GCF_900692235.1_9221_6_26_genomic
GCF_900692255.1_9221_6_28_genomic
GCF_900692265.1_9221_6_36_genomic
GCF_900692275.1_9221_6_35_genomic
GCF_900692285.1_9221_6_33_genomic

GCF_900692295.1_9221_6_38_genomic
GCF_900692305.1_9221_6_41_genomic
GCF_900692315.1_9221_6_40_genomic
GCF_900692325.1_9221_6_37_genomic
GCF_900692335.1_9221_6_42_genomic
GCF_900692345.1_9221_6_39_genomic
GCF_900692355.1_9221_6_43_genomic
GCF_900692365.1_9221_6_44_genomic
GCF_900696735.1_Metronidazole_resistant_Clostridioides_difficile_genomic
GCF_900744365.1_PUC47_genomic
GCF_900744445.1_PUC51_genomic
GCF_900744455.1_PUC99_genomic
GCF_900744495.1_PUC347_genomic
GCF_900744565.1_PUC577_genomic
GCF_901002035.1_4995_2_4_genomic
GCF_901002045.1_4880_7_10_genomic
GCF_901002085.1_4995_2_11_genomic
GCF_901002095.1_4995_2_2_genomic
GCF_901002125.1_4880_5_9_genomic
GCF_901002135.1_4995_2_10_genomic
GCF_901002485.1_4995_1_3_genomic
GCF_901002495.1_4995_1_10_genomic
GCF_901002505.1_4995_1_5_genomic
GCF_901002515.1_4880_3_1_genomic
GCF_901002525.1_4880_3_5_genomic
GCF_901002545.1_4995_2_5_genomic
GCF_901002565.1_4995_1_4_genomic
GCF_901002585.1_4880_3_7_genomic
GCF_901002615.1_4880_5_5_genomic
GCF_901002625.1_4995_1_7_genomic
GCF_901002635.1_4880_3_2_genomic
GCF_901002655.1_4995_2_6_genomic
GCF_901004475.1_7614_4_52_genomic
GCF_901004485.1_7614_4_50_genomic
GCF_901004495.1_7614_4_53_genomic
GCF_901004505.1_7614_4_54_genomic
GCF_901004515.1_7614_4_56_genomic
GCF_901004525.1_7614_4_58_genomic
GCF_901004535.1_7614_4_61_genomic
GCF_901004545.1_7614_4_60_genomic
GCF_901004555.1_7614_4_64_genomic
GCF_901004565.1_7614_4_79_genomic
GCF_901004585.1_7614_4_68_genomic
GCF_901004605.1_7614_4_71_genomic
GCF_901004615.1_7614_4_73_genomic
GCF_901004625.1_7614_4_82_genomic
GCF_901004635.1_7614_4_85_genomic
GCF_901004645.1_7614_4_81_genomic
GCF_901004655.1_7614_4_84_genomic
GCF_901004665.1_7614_4_88_genomic
GCF_901004675.1_7614_4_86_genomic
GCF_901004685.1_7614_4_87_genomic
GCF_901004695.1_7614_4_90_genomic
GCF_901004705.1_7614_4_89_genomic
GCF_901004725.1_7614_4_91_genomic
GCF_901004735.1_7614_7_13_genomic
GCF_901004745.1_7614_7_14_genomic

GCF_901004755.1_7614_7_11_genomic
GCF_901004775.1_7614_7_18_genomic
GCF_901004785.1_7614_7_24_genomic
GCF_901004795.1_7614_7_23_genomic
GCF_901004805.1_7614_7_19_genomic
GCF_901004815.1_7614_7_20_genomic
GCF_901004835.1_7614_7_21_genomic
GCF_901004845.1_7614_7_26_genomic
GCF_901004855.1_7614_7_29_genomic
GCF_901004865.1_7614_7_27_genomic
GCF_901004875.1_7614_7_33_genomic
GCF_901004885.1_7614_7_28_genomic
GCF_901004895.1_7614_7_35_genomic
GCF_901004905.1_7614_7_36_genomic
GCF_901004925.1_7614_7_37_genomic
GCF_901004935.1_7614_7_41_genomic
GCF_901004945.1_7614_7_3_genomic
GCF_901004955.1_7614_7_42_genomic
GCF_901004965.1_7614_7_34_genomic
GCF_901004975.1_7614_7_43_genomic
GCF_901004985.1_7614_7_5_genomic
GCF_901005005.1_7614_7_4_genomic
GCF_901005015.1_7614_7_6_genomic
GCF_901005025.1_7614_7_8_genomic
GCF_901005035.1_7614_7_9_genomic
GCF_901006375.1_8140_6_8_genomic
GCF_901006405.1_8140_6_93_genomic
GCF_901006425.1_8140_6_94_genomic
GCF_901006475.1_8140_6_9_genomic
GCF_901523085.1_45232_E01_genomic
GCF_902386365.1_UHGG_MGYG-HGUT-02369_genomic
GCF_902806655.1_HON10_genomic
GCF_902806665.1_HON06_genomic
GCF_902806675.1_DF11_genomic
GCF_902806705.1_HON11_genomic
GCF_944989955.1_JMR5_assembly_genomic
SF100_assembly_filtered
SF101_assembly_filtered
SF102_assembly_filtered
SF103_assembly_filtered
SF104_assembly_filtered
SF105_assembly_filtered
SF106_assembly_filtered
SF107_assembly_filtered
SF108_assembly_filtered
SF109_assembly_filtered
SF111_assembly_filtered
SF112_assembly_filtered
SF113_assembly_filtered
SF114_assembly_filtered
SF116_assembly_filtered
SF117_assembly_filtered
SF118_assembly_filtered
SF119_assembly_filtered
SF120_assembly_filtered
SF121_assembly_filtered
SF122_assembly_filtered

SF124_assembly_filtered
SF125_assembly_filtered
SF126_assembly_filtered
SF127_assembly_filtered
SF128_assembly_filtered
SF129_assembly_filtered
SF130_assembly_filtered
SF131_assembly_filtered
SF132_assembly_filtered
SF133_assembly_filtered
SF134_assembly_filtered
SF135_assembly_filtered
SF136_assembly_filtered
SF137_assembly_filtered
SF138_assembly_filtered
SF139_assembly_filtered
SF140_assembly_filtered
SF141_assembly_filtered
SF142_assembly_filtered
SF143_assembly_filtered
SF144_assembly_filtered
SF145_assembly_filtered
SF146_assembly_filtered
SF147_assembly_filtered
SF148_assembly_filtered
SF149_assembly_filtered
SF150_assembly_filtered
SF151_assembly_filtered
SF152_assembly_filtered
SF153_assembly_filtered
SF154_assembly_filtered
SF155_assembly_filtered
SF156_assembly_filtered
SF157_assembly_filtered
SF158_assembly_filtered
SF159_assembly_filtered
SF160_assembly_filtered
SF161_assembly_filtered
SF162_assembly_filtered
SF163_assembly_filtered
SF164_assembly_filtered
SF165_assembly_filtered
SF64_assembly_filtered
SF65_assembly_filtered
SF66_assembly_filtered
SF67_assembly_filtered
SF68_assembly_filtered
SF69_assembly_filtered
SF70_assembly_filtered
SF71_assembly_filtered
SF72_assembly_filtered
SF73_assembly_filtered
SF74_assembly_filtered
SF75_assembly_filtered
SF76_assembly_filtered
SF77_assembly_filtered
SF78_assembly_filtered

SF79_assembly_filtered
SF80_assembly_filtered
SF81_assembly_filtered
SF82_assembly_filtered
SF83_assembly_filtered
SF84_assembly_filtered
SF85_assembly_filtered
SF86_assembly_filtered
SF87_assembly_filtered
SF88_assembly_filtered
SF89_assembly_filtered
SF90_assembly_filtered
SF91_assembly_filtered
SF92_assembly_filtered
SF93_assembly_filtered
SF94_assembly_filtered
SF95_assembly_filtered
SF96_assembly_filtered
SF97_assembly_filtered
SF98_assembly_filtered
SF99_assembly_filtered
TN-001-S04_CD_assembly_filtered
TN-002-S01_CD_assembly_filtered
TN-003-S00_CD_assembly_filtered
TN-004-S01_CD_assembly_filtered
TN-005-S01_CD_assembly_filtered
TN-006-S01_CD_assembly_filtered
TN-007-S01_CD_assembly_filtered
TN-008-S01_CD_assembly_filtered
TN-009-S00_CD_assembly_filtered
TN-010-S00_CD_assembly_filtered
TN-010-S04_CD_assembly_filtered
TN-011-S00_CD_assembly_filtered
TN-012-S00_CD_assembly_filtered
TN-012-S04_CD_assembly_filtered
TN-013-S00_CD_assembly_filtered
TN-014-S00_CD_assembly_filtered
TN-015-S00_CD_assembly_filtered
